# Morphogenesis of bacterial colonies in liquid crystalline environments

**DOI:** 10.1073/pnas.2536343123

**Published:** 2026-08-18

**Authors:** Sebastian Gonzalez La Corte, Thomas G. J. Chandler, Saverio E. Spagnolie, Ned S. Wingreen, Sujit S. Datta

**Affiliations:** ^a^ Lewis-Sigler Institute for Integrative Genomics, Princeton University, Princeton, NJ 08544; ^b^ Department of Mathematics, University of North Carolina at Chapel Hill, Chapel Hill, NC 27599-3250; ^c^ Department of Mathematics, University of Wisconsin–Madison, Madison, WI 53706; ^d^ Department of Molecular Biology, Princeton University, Princeton, NJ 08544; ^e^ Division of Chemistry and Chemical Engineering, California Institute of Technology, Pasadena, CA 91125; ^f^ Department of Chemical and Biological Engineering, Princeton University, Princeton, NJ 08544

**Keywords:** nematic, liquid crystal, bacteria, colony growth, active matter

## Abstract

Natural bacterial habitats are often complex fluids with viscoelastic and anisotropic responses to stress; for example, they can take the form of liquid crystals (LCs), with elongated microscopic constituents that collectively align while still retaining the ability to flow. However, laboratory studies typically focus on cells in simple liquids or complex fluids with randomly oriented constituents. Here, we show how interactions with LCs shape bacterial proliferation in multicellular colonies. Using experiments, we find that in a nematic LC, cells of the multiple species we tested all form aligned single-cell-wide “chains” as they reproduce. As these chains lengthen, they eventually buckle in a highly localized manner. By combining our measurements with a continuum mechanical theory, we demonstrate that this distinctive morphogenetic program emerges because cells are kept in alignment due to the LC’s elasticity; as each chain lengthens, growth-induced viscous stresses along its contour eventually overcome the elasticity of the surrounding nematic, leading to buckling. Our work thus reveals and provides mechanistic insight into the previously overlooked role of LCs in sculpting bacterial life in complex environments.

## Significance

Many bacteria live in environments with the characteristics of liquid crystals—fluids whose molecules are elongated and locally align in the same direction. How this alignment affects bacterial colonies remains unknown. We show that when nonmotile bacteria reproduce in a liquid crystal, they form long, aligned chains that eventually buckle in a distinctive localized pattern. This colony morphology arises from competition between the liquid crystal’s anisotropic elasticity, which keeps cells aligned, and growth-induced stresses that trigger buckling. This work reveals how physical interactions with complex environments can sculpt bacterial life, potentially influencing interactions with nutrients and antibiotics. It also demonstrates that when particles can proliferate, liquid crystalline environments promote new forms of self-organization, enabling engineered living materials with controlled architectures.

Many bacterial habitats can exhibit liquid crystalline (LC) order; prominent examples include host mucus (1–3), extracellular polymeric substances in biofilms (4), and dense suspensions of filamentous phages (5–7). However, laboratory studies of bacteria typically focus on cells in isotropic environments. As a result, whether and how inhabiting a liquid crystal influences bacterial behavior remains poorly understood.

Recent work indicates that the anisotropic rheology of LCs can dramatically alter how individual cells swim (8–15) and aggregate with other cells (8, 11, 16–21). Nevertheless, despite growing recognition that an environment’s mechanical properties, in addition to its chemistry, can influence bacterial growth (22–27), the possible influence of liquid crystalline order on another fundamental characteristic of bacterial life—proliferation through space in a multicellular colony (28)—remains underexplored. Addressing this gap in knowledge is important to establish mechanistic principles governing how proliferating active matter interacts with complex environments (26, 29). It also has key biological implications; indeed, in nature, many bacteria are nonmotile or lose motility (30–34), but continue to proliferate in colonies. Colony morphology in turn influences how cells access nutrients, cooperate and compete with each other, resist external stressors (e.g., antibiotics, predators, phage), and cause infections in hosts (27, 28, 35–52). Hence, we ask: Can an environment’s liquid crystalline order affect the morphology of proliferating bacterial colonies?

Here, we demonstrate that this is indeed the case, and we elucidate the underlying biophysical mechanisms. Through experiments, we find that rod-shaped nonmotile bacteria form single-cell-wide “chains” as they proliferate in a nematic LC, all aligned along the LC director—in stark contrast to forming a random dispersion as in the conventionally studied non-LC case. The chains elongate as proliferation continues, eventually buckling after reaching a critical length. This mechanical instability occurs over a much smaller length scale within the chain than the typical Euler buckling exhibited by long, slender filaments under compression. Using a continuum mechanical theory, we trace the origin of chain formation and buckling to the competition between LC elasticity, which tends to keep cells aligned along the LC director, and growth-induced compression from frictional stresses along each lengthening chain. This work thereby reveals how liquid crystalline environments can dramatically shape proliferating bacterial colonies and provides quantitative principles to predict and control these morphodynamics more broadly.

## Experimental Results

### Nonmotile Bacteria Form Long, Single-Cell-Wide Chains as They Proliferate in a Nematic Liquid Crystal.

**Fig. 1.**
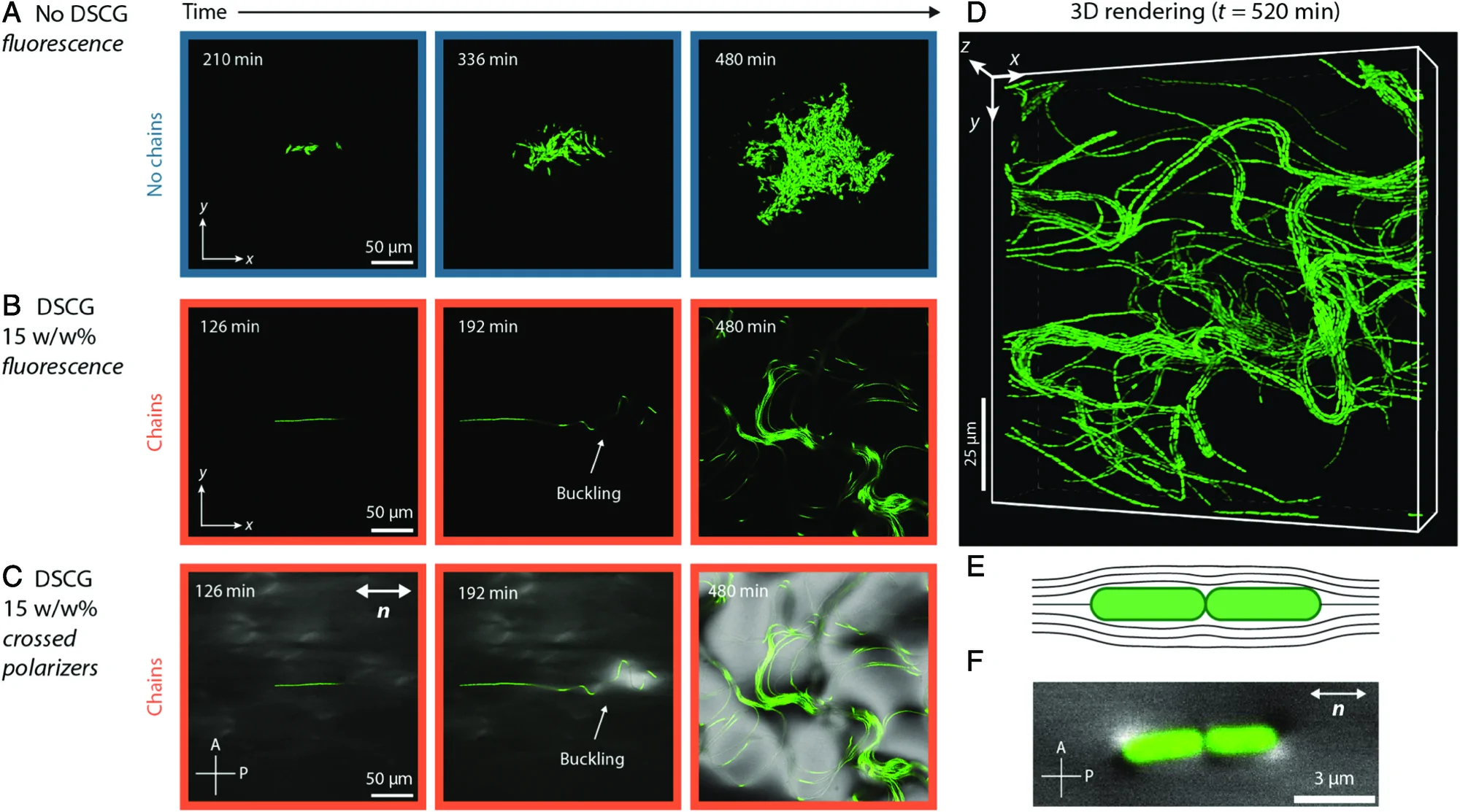
Proliferating nonmotile bacteria form chain-like colonies in liquid crystalline environments. (*A*) Fluorescent confocal microscopy time sequence of nonmotile *E. coli* proliferating in nutrient-rich LC-free fluid; cells form an isotropic dispersion as they grow, divide, and diffuse apart. (*B*) Same as in (*A*), but in nematic DSCG solution (15 w/w%); cells instead remain aligned end-to-end, forming a single-cell-wide chain that eventually buckles. (*C*) Same as in (*B*) with crossed-polarizer imaging overlaid (directions indicated by A and P), revealing local reorientation of the LC director field n accompanying chain buckling (bright regions). The overall far-field orientation imposed on the director by the underlying substrate is shown by the double-headed arrow. (*D*) Three-dimensional confocal reconstruction of a chain-like colony after 520 min, showing the serpentine, single-cell-wide internal structure. (*E*) Schematic of the LC director profile around a rod-shaped bacterium with weak tangential anchoring; boojums (topological defects) form at the cell poles where the director field must accommodate the curved cell surface. (*F*) Fluorescent confocal micrograph with crossed-polarizer imaging overlaid (directions indicated by A and P) of two cells dispersed in nematic DSCG solution. The image shows the local reorientation of the LC director field n around the cell, with the emergence of boojums at the cell poles. The overall far-field orientation imposed on the director by the underlying substrate is shown by the double-headed arrow. Experiments showed are performed using a microfluidic device 25 μm in height, 25 mm in width, and 22 mm in length.

Our experiments probe the proliferation of nonmotile bacteria in nutrient-rich aqueous solutions housed in thin, flat polydimethylsiloxane (PDMS) imaging channels bonded to underlying glass substrata. To start, we use *Escherichia coli* as a model bacterium; the cells constitutively express green fluorescent protein (GFP) in their cytoplasm, enabling direct visualization using fluorescence confocal microscopy. In LC-free solution, the cells continually grow, divide, separate from each other, and diffuse apart, eventually forming an isotropic dispersion (Fig. 1*A* and Movie S1).

We observe dramatically different behavior upon addition of disodium chromoglycate (DSCG), a nontoxic lyotropic LC (53, 54), to the solution at a concentration c=15w/w%. Under these conditions, the solution forms a nematic (55), in which the chromonic DSCG molecules self-assemble into elongated stacks that tend to collectively align along the same direction (56), which we prescribe by rubbing the glass substrate with a diamond lapping film (57). In this case, instead of becoming randomly oriented, cells are retained end-to-end as they proliferate, forming single-cell-wide “chains” aligned along their long axis in the direction of LC alignment (Fig. 1*B*, first frame). As proliferation progresses, the chains elongate, eventually buckling (second frame)—bending the surrounding LC in turn, as shown by the local reorientation of the LC director field n (Fig. 1*C* and Movie S2), which we visualize using crossed polarizers placed around the sample in the microscope’s light path. Intriguingly, the chains form multiple, highly localized, sharply curved arcs as they buckle (arrows, second frame of Fig. 1 *B* and *C*)—in stark contrast to the classic Euler buckling instability of a compressed long, slender filament, which instead results in the formation of a single, long-wavelength arc. The chains grow in close proximity to either the top or bottom surface, often with at least one endpoint transiently pinned to it (*SI Appendix*); as they continue to elongate, these localized buckles continue to grow near the surface and new buckling events arise as well (Movie S2), eventually creating an intertwined three-dimensional network of long, serpentine, multicellular, single-cell-wide chains (Fig. 1*D*).

To test the generality of this phenomenon, we perform the same experiment using nonmotile strains of two other species of bacteria: *Vibrio cholerae* and *Pseudomonas aeruginosa*. We again observe chain formation in both cases (*SI Appendix*, Fig. S5 *A* and *B*), indicating that this phenomenon arises across different cell types. Together, these results demonstrate that nonmotile bacterial colonies proliferating in nematic LCs consistently form large, serpentine, single-cell-wide chains.

### Nematic Elasticity Causes Chain Formation.

Why do chains form? One possibility is that interactions with the surrounding LC alter how the individual cells grow, somehow causing them to proliferate in chains. However, bacterial growth rates directly measured using single-cell microscopy show no appreciable differences upon LC addition to the solution (*SI Appendix*, Fig. S4 *A* and *B*). Moreover, the osmotic pressure of the LC is ≈2kPa (*SI Appendix*), far lower than the pressure at which bacterial physiology is typically altered, ∼100kPa (58–61)—further arguing against this possibility.

What other features of the LC drive chain formation? Close inspection of the LC director field n around a pair of cells during the nascent stage of chain formation provides a clue. The cells orient end-to-end along the imposed direction of LC alignment, with two opposing pairs of bright and dark regions flanking their outer poles apparent in the crossed polarizer micrograph (Fig. 1 *E* and *F*). These features are known as “boojums,” topological defects that form at the cell poles where the tangentially anchored nematic director field must accommodate the curved spherocylindrical cell (62, 63). The emergence of these point defects at the poles, rather than ring defects encircling the cell or complete disruption of the nematic order, indicates that the LC is tangentially anchored to the cell surface with a finite anchoring strength (8, 64). That is, the elongated constituents of the LC tend to collectively align along both the direction imposed on the glass substrate as well as the surfaces of the cells.

Deviations from this preferred alignment cost energy; following the so-called one-constant approximation, all such deviations are penalized by a single Frank elastic constant, K. A minute rotation of the cell’s long axis away from the overall imposed direction of the nematic director by an angle $\theta =2^{{}^{\circ}}$ requires $U_{\text{el}}\approx 2\pi \left[l_{b}/\;\text{log}\left(2l_{b}/a\right)\right]K\theta^{2}∼500\;k_{\text{B}}T$ in energy (8, 64, 65), where $l_{b}$ and a are the cell length and cross-sectional radius, respectively—far greater than the thermal energy $k_{\text{B}}T$ (*SI Appendix*). Therefore, the nematic elasticity of the LC forces cells to strongly align with it (8, 64) and continue to proliferate as aligned chains.

**Fig. 2.**
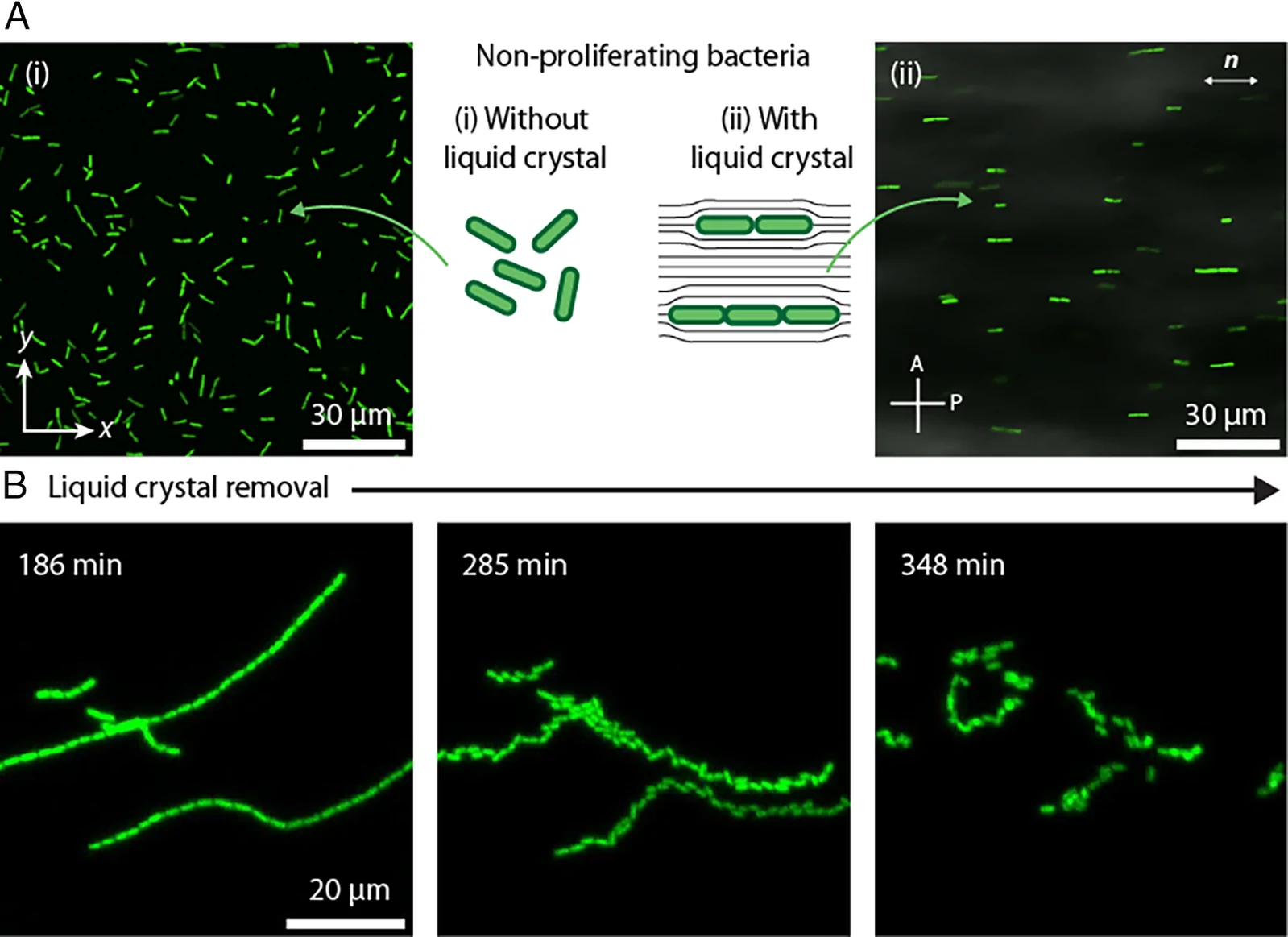
LC-mediated elastic forces are required for chain formation. (*A*) Nonproliferating *E. coli* cells (*i*) remain randomly dispersed in LC-free fluid but (*ii*) spontaneously align and form end-to-end aggregates in nematic DSCG (18 w/w%). Images show confocal micrographs with crossed-polarizer imaging overlaid in (*ii*) as in Fig. 1 *C* and *F*. (*B*) Time sequence showing chain disintegration after LC removal; LC-free solvent is introduced at t=0. Cells remain in smaller side-by-side aggregates after LC removal, likely due to residual surface adhesion interactions (66, 67) that persist after cells are brought together by LC-induced elastic forces. See Movie S3 for full field of view and dynamics.

Two additional sets of experiments confirm this picture. First, we culture cells in nutrient-free solutions in which proliferation is arrested. When the solution does not contain LC, the cells are disconnected and randomly dispersed, with no orientational order (Fig. 2*A*, *i*); by contrast, in the LC, the cells orient along the imposed direction of LC alignment (Fig. 2*A*, *ii*), consistent with our expectation and with previous observations of elongated ellipsoidal colloids in a nematic LC (68). Second, given that the nematic elasticity driving chain formation is generated by the LC, we expect that after a chain has formed, removing the LC from the solution around it should allow the chain to disintegrate via random thermal motion of the cells. This is precisely what we observe upon flushing a suspension of chains with LC-free liquid (Fig. 2*B* and Movie S3). Taken altogether, these observations establish that nematic elasticity induced by the LC causes chains to form.

### Chains Buckle upon Reaching a Critical Length.

**Fig. 3.**
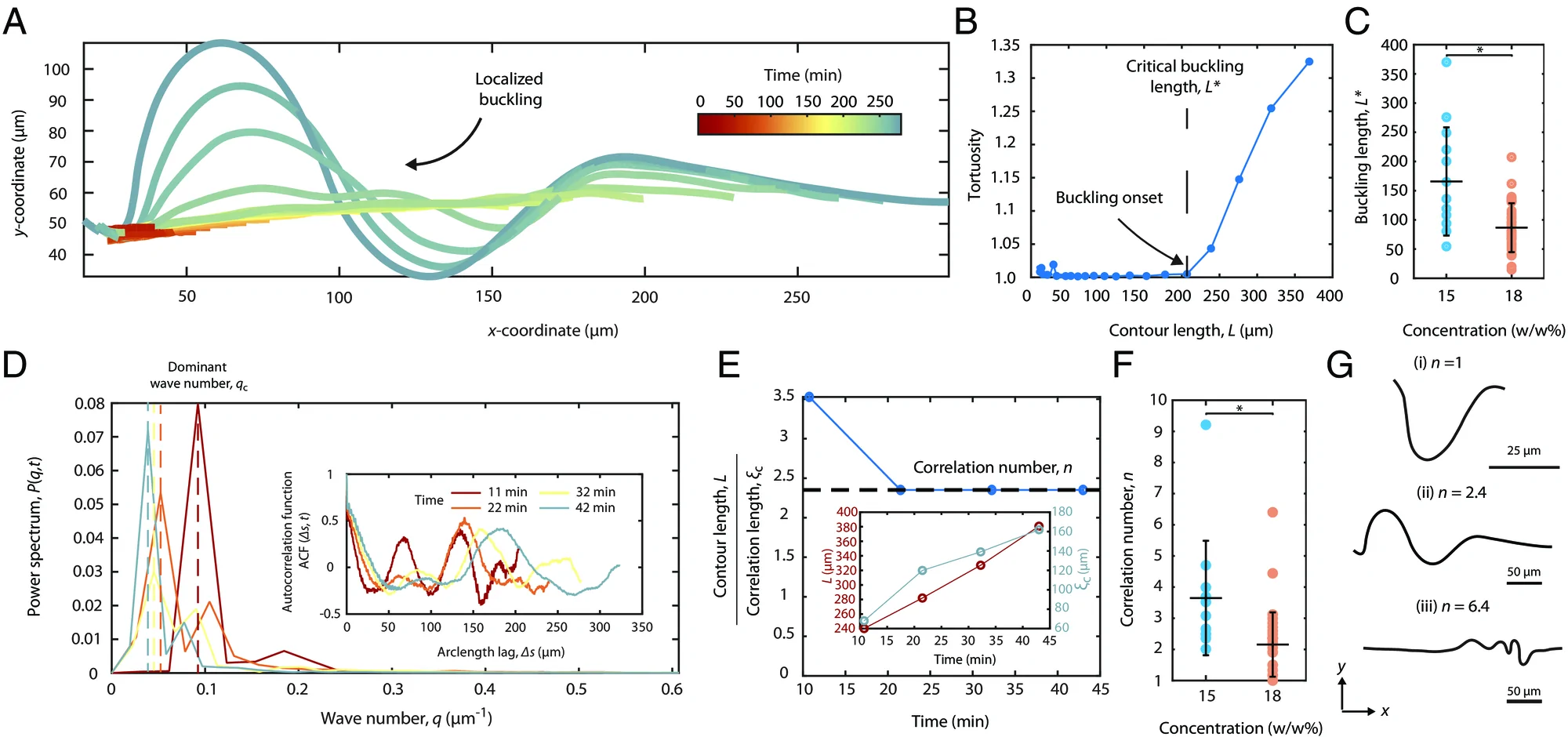
Characterization of chain buckling. (*A*) Successive centerline configurations of a buckling chain in 18 w/w% DSCG, with colors indicating time. The chain initially grows straight before developing highly localized, sharply curved buckles. (*B*) Tortuosity (contour length divided by end-to-end distance) as a function of contour length for the chain in (*A*); the sharp increase marks the onset of buckling at the critical length $L^{*}$. (*C*) Measured buckling lengths for chains in 15 w/w% (light blue, n=13) and 18 w/w% (light orange, n=38) DSCG. Horizontal lines indicate means; the critical buckling length decreases with increasing LC concentration (* indicates P<0.05, two-sample t test). (*D*) Power spectrum P(q,t) of the chain in (*A*) at multiple time points after buckling onset. The dashed lines mark the dominant wavenumber $q_{\text{c}}$ where P is maximal; the *Inset* shows the corresponding curvature autocorrelation function ACF(Δs,t). We define the correlation length as $\xi_{\text{c}}\left(t\right)\equiv 2\pi /q_{\text{c}}\left(t\right)$. (*E*) Time evolution of the correlation number $n\equiv L/\xi_{\text{c}}$ for the chain in (*A*); although L and $\xi_{c}$ individually grow (*Inset*), their ratio saturates at n≈2.4. (*F*) Measured correlation numbers for the same chains as in (*C*); horizontal lines indicate means (* indicates P<0.05, two-sample t test). (*G*) Representative centerline configurations of buckling chains with distinct correlation numbers: Chains with both endpoints firmly pinned to the top or bottom surface exhibit lower n approaching ≈1 (a single long-wavelength arc), while chains with only transient single-end pinning develop higher n with multiple localized buckles. High-curvature regions occupy progressively smaller fractions of the contour as n increases.

Having established the origin of chain formation, we next ask: Why do chains buckle as their constituent cells continue to proliferate (Fig. 1 *B* and *C*)? And why does their buckling appear to be so different from the classic Euler buckling instability? To begin to address these questions, we first examine our experimental observations of chain buckling. Snapshots of the contour of a representative chain as it elongates are shown in Fig. 3*A*; it is initially straight, but buckles, becoming more tortuous, after elongating sufficiently. Direct measurement of the chain’s tortuosity, defined as its contour length L normalized by its end-to-end distance, as it grows quantifies this observation: As shown in Fig. 3*B*, the tortuosity increases sharply as the chain elongates beyond a length ≈200μm. We denote this critical buckling length by $L^{*}$. Repeating this measurement for many other chains indicates that they consistently buckle at a critical length that decreases with increasing LC concentration (i.e., increasing nematic elasticity): $L^{*}=170\pm 90\;\mu m$ and 90±40μm for experiments performed using c=15w/w% and 18w/w%, respectively (Fig. 3*C*). In all cases, the buckling is far more localized than the long-wavelength arc formation characteristic of Euler buckling, as in Fig. 1 *B* and *C*.

To quantify the spatial structure of the buckled chains, we examine the autocorrelation of their local curvature along the contour. Specifically, for each chain at each time point after the onset of buckling, we compute the unbiased spatial autocorrelation function of the normalized local curvature vector κ(s,t), $\text{ACF}\left(\Delta s,t\right)={\left(s_{\text{max}}-\Delta s\right)}^{-1}\int_{0}^{s_{\text{max}}-\Delta s}\kappa \left(s,t\right)\cdot \kappa \left(s+\Delta s,t\right)\;\;\text{d}s$, where s is the arclength along the chain centerline, t is the time, Δs is the arclength lag, and $s_{\text{max}}$ is restricted to 75 to 90% of the total contour length to avoid edge artifacts. The ACF decays rapidly at small lags and develops local maxima at intermediate lags set by the spacing between high-curvature regions (Fig. 3*D*, *Inset*). To extract the characteristic spatial scale of this periodicity and track its temporal evolution, we compute the corresponding power spectrum, $P\left(q,t\right)=|s_{\text{max}}^{-1}\int_{0}^{s_{\text{max}}}\left[\;\text{ACF}\left(\Delta s,t\right)-\bar{\text{ACF}}\left(t\right)\right]\;e^{-iq\Delta s}\;\;\text{d}\Delta s{|}^{2}$, where $\bar{\text{ACF}}\left(t\right)$ denotes the mean of the ACF profile at time t. While the power is distributed across several wavenumbers, a distinct peak emerges at a dominant wavenumber $q_{\text{c}}\left(t\right)$ (Fig. 3*D*), from which we define the correlation length $\xi_{\text{c}}\left(t\right)\equiv 2\pi /q_{\text{c}}\left(t\right)$. The ratio of the contour length to the correlation length, which we term the correlation number $n\equiv L/\xi_{\text{c}}$, then quantifies how many correlated intervals fit within the chain. Although L and $\xi_{\text{c}}$ both grow as the chain elongates after the onset of buckling, their ratio saturates at n≈2.4 for the representative chain shown in Fig. 3*E*. Indeed, we measure n>1 for the majority of tracked chains in our experiments (Fig. 3 *F* and *G* and *SI Appendix*), indicating that localized buckling—in stark contrast to the classical Euler buckling of compressed slender filaments, for which the unstable mode spans the entire filament so that n∼1—is a robust feature of bacterial proliferation in nematic LCs.

## Mathematical Modeling

### Force Balance of a Growing Continuum Chain.

**Fig. 4.**
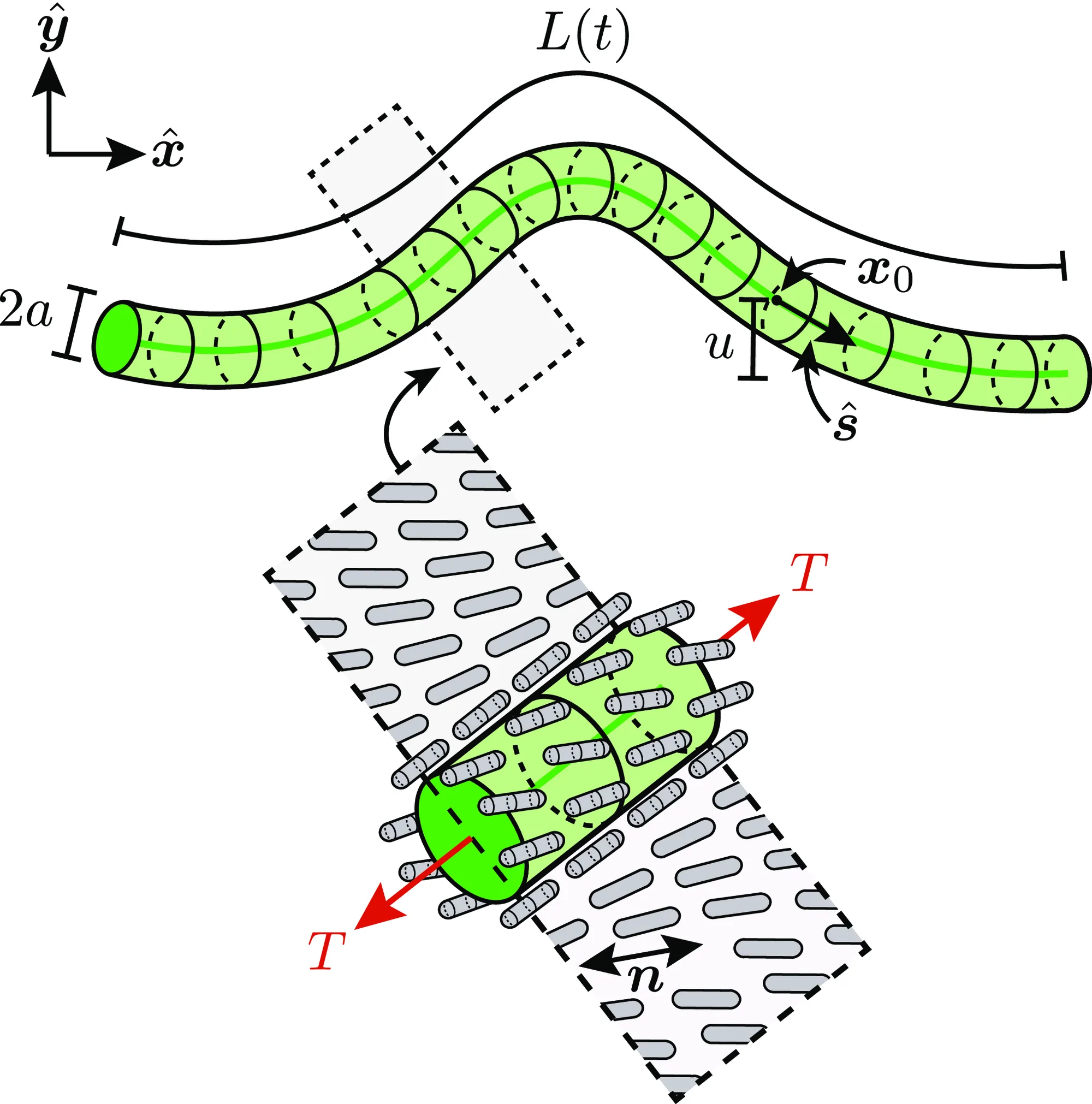
Continuum model of a growing bacterial chain in a nematic LC. The chain is treated as a slender filament of fixed radius a and growing length L(t). The transverse ($\hat{y}$) displacement u(s,t) is parameterized by the arclength s. Growth generates internal compression T(s,t) along the chain contour due to viscous drag from the surrounding LC, while chain deflections are resisted by the elastic restoring force from the deformed nematic director field (gray rods, *Bottom*).

Our experiments highlight the pivotal role of nematic elasticity in causing chains to form and influencing how they buckle. Indeed, while the bacterial chains are composed of discrete, unattached cells, the nematic elasticity of the surrounding LC effectively couples adjacent cells together—causing deformations of a given chain to be mechanically correlated over distances much larger than a single cell length. Hence, to shed light on the physics underlying chain growth and buckling, we turn to mathematical modeling. In particular, we describe each chain as a continuous, slender, elastic filament (Fig. 4) of fixed radius a and a length that grows exponentially in time t, $L\left(t\right)=L\left(0\right)e^{\alpha t}$, where α is the single-cell growth rate. The chain centerline is parameterized by the arclength s∈[−L(t)/2,L(t)/2] as $x_{0}\left(s,t\right)$, with centerline unit tangent vector $\hat{s}=\partial_{s}x_{0}$. The material velocity at each point, V(s,t), can be decomposed as $V=\alpha s\hat{s}+v\left(s,t\right)$, where the first term reflects tangential growth.

The force per unit length acting on the chain at each station (point along the centerline) s is then given by

[1] $$\begin{array}{c} f\left(s,t\right)=f^{\text{viscous}}\left(s,t\right)+f^{\text{LC}}\left(s,t\right)+\partial_{s}\left[T\left(s,t\right)\hat{s}\right], \end{array}$$

where $f^{\text{viscous}}$ is the viscous drag per unit length induced by the LC, $f^{\text{LC}}$ is the elastic restoring force per unit length from the deformed nematic, and T is the internal tension (or compression if T<0) arising from bacterial growth. Because viscous damping is much larger than inertial effects in our experiments, f=0 at all times (69); since the bacterial chain grows very slowly compared to the other time scales governing the LC flow, the LC is assumed to be in quasi-static equilibrium at all times. Moreover, because the chain is slender, we can use the resistive force approximation, which assumes the drag at each point depends only on the local chain velocity and orientation, to calculate $f^{\text{viscous}}=-\left[\zeta_{\|}\hat{s}\hat{s}+\zeta_{⊥}\left(\text{I}-\hat{s}\hat{s}\right)\right]\cdot V$; the drag coefficients $\zeta_{\|}$ and $\zeta_{⊥}$ penalize motion in the chain tangent and normal directions, respectively. Eq. **1** can then be decomposed into tangential and normal components as

[2a] $$\begin{array}{c} -\zeta_{\|}\left(v_{\|}+\alpha s\right)+\partial_{s}T=0, \end{array}$$

[2b] $$\begin{array}{c} -\zeta_{⊥}v_{⊥}+\left(\text{I}-\hat{s}\hat{s}\right)\cdot \left(f^{\text{LC}}+T\partial_{s}\hat{s}\right)=0, \end{array}$$

where the material velocity $v=v_{\|}\hat{s}+v_{⊥}$ with $\hat{s}\cdot v_{⊥}=0$, and $\hat{s}\cdot f^{\text{LC}}=0$ (detailed in *SI Appendix*). Assuming small chain displacements for simplicity, we approximate the deformed position as $x_{0}\left(s,t\right)\approx s\hat{x}+u\left(s,t\right)\hat{y}$, where $\hat{x}$, $\hat{y}$, and $\hat{z}$ denote the unit Cartesian vectors and u is the vertical displacement. We then have that $v_{⊥}\approx \partial_{t}u\hat{y}$ and $\hat{s}\approx \hat{x}+\partial_{s}u\hat{y}$, giving

[3] $$\begin{array}{c} \zeta_{⊥}\partial_{t}u=T\left(s,t\right)\partial_{\mathrm{ss}}u+\hat{y}\cdot f^{\text{LC}}\left(s,t\right). \end{array}$$

To close this system of equations, we compute the tension T and LC restoring force $f^{\text{LC}}$ for the two principal cases that arise in the experiments: When the chain is i) free at both ends (“free–free”) or ii) fixed at one end, e.g., due to pinning on the glass substrate and free at the other (“fixed-free”). Details are provided in *SI Appendix*.

In brief, integrating Eq. **2a** yields the tension T(s,t), which depends on α, $\zeta_{\|}$, L(t), and the endpoint tension $T_{L}$. In both cases i) and ii), as growth progresses, the tension eventually becomes negative (i.e., compressive)—introducing the possibility of buckling (70). However, unlike in classical Euler buckling, the load is distributed *along* the length of the chain due to the viscous drag, just as in the buckling of sedimenting filaments (71) and filaments placed in compressional flows (72–74). To compute the LC elastic restoring force, we assume that the LC is deep in the nematic state with director field n(x,t) and |n|=1 (75). With $n\to \hat{x}$ as |x|→∞, we assume for simplicity that $n\left(x\right)\approx \hat{x}+p\left(x\right)$ with $\hat{x}\cdot p=0$, for a small perturbation in the molecular orientation $p=O(\partial_{s}u)$. Using the one-constant approximation for the Frank elastic energy, $\nabla^{2}p=0$ (75), enabling us to compute the LC traction (surface force per unit area) acting on the chain. We thereby compute $f^{\text{LC}}\left(s,t\right)$, which depends on K, a, L(t), and the anchoring strength W quantifying the energetic penalty for LC molecules to deviate from tangential alignment with the chain surface.

### Parameters Characterizing Bacterial Chain Growth in a Liquid Crystal.

To distill the relevant physics from our theoretical model, we first reduce the number of parameters in Eqs. **1**–**4** by scaling lengths and time by the constant chain radius a and inverse growth rate $\alpha^{-1}$, respectively. Using these natural scales, we obtain four key dimensionless parameters that govern chain growth:

- The chain aspect ratio, Λ≡L/(2a), which increases to ≫1 as growth progresses.
- The drag anisotropy ratio, $\chi \equiv \zeta_{⊥}/\zeta_{\|}$. Approximating the chain as a filament moving in an isotropic fluid a distance h from a surface, with h≪L (76), $\zeta_{\|}\approx \left[2\pi /\log\left(2h/a\right)\right]\mu_{\|}$ and $\chi \equiv \zeta_{⊥}/\zeta_{\|}\approx 2\mu_{⊥}/\mu_{\|}$ for the LC perpendicular and parallel viscosities $\mu_{⊥}$ and $\mu_{\|}$, respectively. Typical viscosity ratios for DSCG range from $\mu_{⊥}/\mu_{\|}\approx 2$ to 6 (77, 78), so we fix χ≈5.
- The dimensionless anchoring strength, w≡aW/K. We estimate that our experiments probe $w∼10^{-2}$ to $10^{0}$ (8, 79, 80) (*Materials and Methods*), consistent with the weak tangential anchoring indicated by the micrograph in Fig. 1*F*.
- The Ericksen number, $\text{Er}\equiv a^{2}\zeta_{\|}\alpha /\left(2\pi K\right)$, which compares viscous forces introduced by chain growth to elastic forces arising from LC deformation. We estimate (*Materials and Methods*) that our experiments probe $\text{Er}\approx 3.0\times 10^{-7}$ (c=15w/w%) and $7.7\times 10^{-7}$ (c=18w/w%). Er≪1, which highlights the dominant role of nematic LC elasticity in controlling chain growth.

The physical parameters underlying these dimensionless parameters and their measured values are summarized in *SI Appendix*, Table S1. As also detailed in *SI Appendix*, we neglect the endpoint tension $T_{L}$ because it is orders of magnitude smaller than the viscous stress $a^{2}\zeta_{\|}\alpha$, thereby simplifying our system of equations further.

### Competition Between LC Elasticity and Growth-Induced Stresses Along the Chain Contour Drives Localized Buckling.

Having nondimensionalized the governing equations, we predict if and when a growing chain will buckle by performing a linear stability analysis of the straight chain state (detailed in *SI Appendix*). That is, we ask: At what chain length (or equivalently, aspect ratio Λ) does a small perturbation to the straight configuration begin to grow rather than decay? To address this question using our model, we perturb the chain centerline with a small displacement $u=ae^{\sigma \tau }U\left(\xi \right)$ (Fig. 4), where ξ≡2s/L(t)∈[−1,1] and τ≡αt; here, σ is the growth rate of the perturbation. We then expand the nondimensional displacement U(ξ,τ) as a Fourier series with time-dependent coefficients and substitute into the dimensionless form of Eq. **3**, with Λ(τ) assumed to be frozen at its instantaneous value ≈Λ. This procedure yields an eigenvalue problem for the growth rate $\sigma_{k}$ of each Fourier mode ${\hat{U}}_{k}$ as a function of Λ:

[4] $$\begin{array}{c} \chi \sigma_{k}{\hat{U}}_{k}=\left(\frac{\chi }{2}+\frac{q_{k}^{2}}{3}-\frac{1}{4}-\frac{q_{k}^{3}}{\text{Er}\;\Lambda^{3}Z_{k}}\right){\hat{U}}_{k}+\sum_{m\neq k}J_{\mathrm{km}}{\hat{U}}_{m}, \end{array}$$

for the free–free case, where $Z_{k}$ quantifies how strongly the LC resists chain deformations at mode k (with $q_{k}\equiv k\pi /2$, so that mode k has wavelength 2L/k) and $J_{\mathrm{km}}$ are coupling coefficients between modes, both derived in *SI Appendix*. The fixed-free case yields an analogous eigenvalue problem with modified mode functions and coupling coefficients. Chain buckling then occurs when any of the modes becomes unstable ($\sigma_{k}>0$), which defines the critical buckling aspect ratio $\Lambda^{*}$ corresponding to the critical length $L^{*}$.

**Fig. 5.**
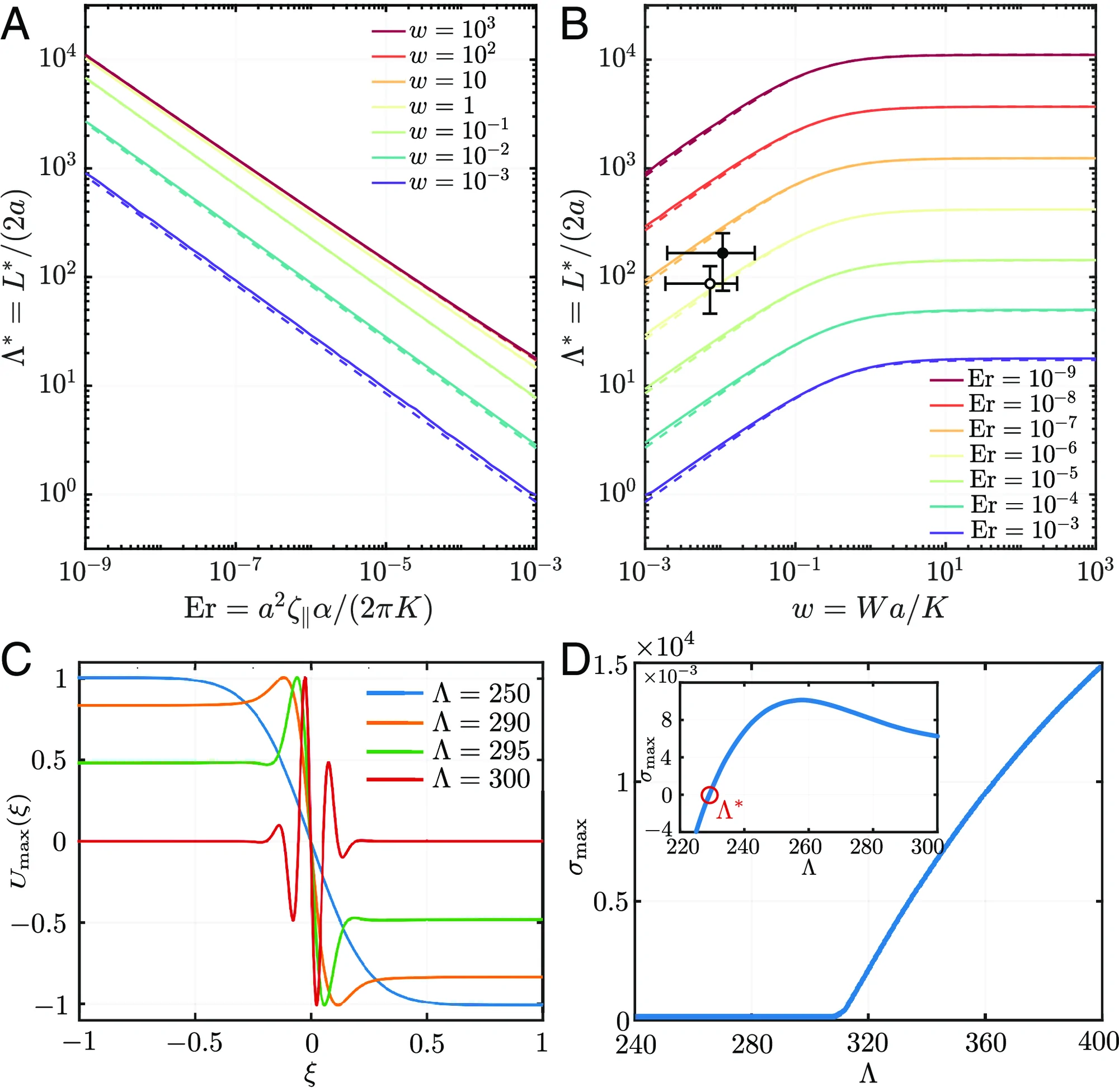
Theoretical predictions for the critical buckling length. (*A*) Dimensionless critical buckling length $\Lambda^{*}\equiv L^{*}/\left(2a\right)$ as a function of the Ericksen number $\text{Er}\equiv a^{2}\zeta_{\|}\alpha /\left(2\pi K\right)$ for different values of the dimensionless anchoring strength w≡aW/K. Solid curves show numerical solutions of the linear stability analysis (Eq. **4**); dashed curves show the analytical approximation (Eq. **5**). The scaling $\Lambda^{*}∼\;{\text{Er}}^{-1/2}$ reflects the balance between growth-induced viscous compression and LC elastic restoring forces. (*B*) $\Lambda^{*}$ as a function of w for different values of Er. For weak anchoring (w≪1), the director can slip relative to the bacterial cell surface, reducing the effective elastic coupling and allowing buckling at shorter chain lengths; for strong anchoring (w≫1), the coupling saturates and $\Lambda^{*}$ becomes independent of w. Symbols show experimentally measured buckling lengths at 15 w/w% (filled circle) and 18 w/w% (open circle) DSCG, plotted at their corresponding Er values; comparison with theory yields inferred anchoring strengths w≈0.01, corresponding to $W\approx 2.6\times 10^{-7}\;J/\;{\text{m}}^{2}$. Both (*A* and *B*) show predictions for the free–free boundary conditions and χ≈5 (*Materials and Methods*). (*C*) The most unstable eigenmode, $U_{\text{max}}\left(\xi \right)$, for χ=5, $\text{Er}=10^{-6}$, w=0.1, and free–free boundary conditions, at four different filament lengths beyond $\Lambda^{*}$. The shape of the first mode to become unstable has a single long wavelength. For longer filaments, the most unstable mode is highly oscillatory and spatially localized near the filament center, akin to the shapes observed in the experiments (e.g. in Fig. 1*C*). (*D*) The growth rate, $\sigma_{\text{max}}$, corresponding to the most unstable eigenmode, is nonmonotonic in the filament length, for lengths between $\Lambda^{*}$ and those at which localized oscillatory modes appear.

Our numerical solutions of $\Lambda^{*}$ from Eq. **4** are shown in Fig. 5 (solid curves) for the exemplary free–free case. The results for the fixed-free case are similar in magnitude, but shifted slightly lower by a factor of ≈1.5 because a fixed end cannot relieve growth-induced compression by moving outward, unlike a free end—which is reflected in differences in T(s,t) between the two cases (*SI Appendix*). Inspired by the solution when the Fourier modes are decoupled ($J_{\mathrm{km}}=0$), we also approximate our results as

[5] $$\begin{array}{c} \Lambda^{*}\approx \sqrt{\frac{a_{1}\;{\text{Er}}^{-1}}{\log(a_{2}\;{\text{Er}}^{-1})+4/w}}, \end{array}$$

which provides a useful analytical expression for $\Lambda^{*}\left(\;\text{Er},w\right)$, shown by the dashed curves; here, $\left\{a_{1},a_{2}\right\}$ are numerically fitted constants equal to {2.91,16.2} and {1.26,0.664} for the free–free and fixed-free cases, respectively, with χ=5. Varying w between $10^{-2}$ and 1, with χ=5 and $\text{Er}=10^{-6}$ fixed, gives predicted buckling lengths in the range of $\Lambda^{*}\in \left[88,375\right]$ (for free–free) and $\Lambda^{*}\in \left[55,269\right]$ (for fixed-free). Decreasing χ from 6 to 2, with w=0.1 and $\text{Er}=10^{-6}$ fixed, gives predicted buckling lengths in the range of $\Lambda^{*}\in \left[217,270\right]$ (for free–free) and $\Lambda^{*}\in \left[149,174\right]$ (for fixed-free).

To test our theoretical predictions, we compare the measured critical buckling lengths (Fig. 3*C*) with those predicted by our theory. As shown by the symbols in Fig. 5*B*, which indicate our measured values at their corresponding Ericksen numbers ($\text{Er}\approx 3.0\times 10^{-7}$ and ≈7.7 × 10^−7^), we find good agreement with the free–free theoretical predictions with dimensionless anchoring strengths $w\approx 1.1\times 10^{-2}$ and $0.73\times 10^{-2}$, respectively; comparing to the fixed-free prediction instead yields slightly larger values, $w\approx 3.0\times 10^{-2}$ and $2.0\times 10^{-2}$, respectively. These values correspond to dimensional anchoring strengths W≈2.6 to $2.8\times 10^{-7}\;J/\;{\text{m}}^{2}$ and 7.2 to $7.6\times 10^{-7}\;J/\;{\text{m}}^{2}$, respectively; because our experiments likely include chains with a mixture of boundary conditions, the true anchoring strength likely lies within these ranges (*SI Appendix*). Nevertheless, these values are in reasonable agreement with previous studies of bacteria and similar inclusions in nematic LCs, which found $W\approx 10^{-7}$ to $10^{-5}\;J/\;{\text{m}}^{2}$ (8, 79, 80).

The consistency between theory and experiment supports our central physical picture: That the competition between LC nematic elasticity and growth-induced viscous stresses along the chain contour drives buckling. Fig. 5 reveals how these two effects control the critical buckling length. Consider first the dependence on the Ericksen number, $\text{Er}\equiv a^{2}\zeta_{\|}\alpha /\left(2\pi K\right)$. The compressive viscous drag force $∼\zeta_{\|}\alpha L^{2}$ increases as the chain grows, eventually exceeding the critical LC elastic restoring force ∼K at $L=L^{*}$. Balancing these forces at buckling yields $\Lambda^{*}∼\;{\text{Er}}^{-1/2}$, as shown by the curves in Fig. 5*A*; larger Er (faster growth or weaker LC elasticity) leads to buckling at shorter chain lengths. At the small Ericksen numbers relevant to our experiments ($\text{Er}∼10^{-7}$), LC elasticity dominates, permitting chains to grow to hundreds of cells before the accumulated compression triggers a buckling instability.

Next, consider the dependence on anchoring strength. For weak anchoring (w≪1) such as in our experiments ($w∼10^{-2}$), the director can slip relative to the bacterial cell surface, reducing the effective elastic coupling between chain deflections and the surrounding LC. This weakened coupling lowers the restoring force resisting buckling, which thus occurs at a relatively low accumulated compressive stress and thus for shorter chains—hence, in this regime, $\Lambda^{*}$ grows with w, as shown in Fig. 5*B*. By contrast, for strong anchoring (w≫1), the LC director couples to the bacterial surface tightly and the elastic coupling saturates; further increases in w provide no additional coupling, so $\Lambda^{*}$ becomes independent of w, as also shown in Fig. 5*B*.

The theoretical analysis also explains why chain buckling differs so strikingly from classical Euler buckling. In Euler buckling, an end-applied compressive load destabilizes a single, long-wavelength mode spanning the entire filament. For a bacterial chain in an LC, by contrast, compression is not applied at the endpoints but is generated by viscous drag continuously along the chain as it grows. Simultaneously, the surrounding nematic acts as a distributed elastic foundation, penalizing deflections at all wavelengths. Together, these effects promote instability at shorter wavelengths, producing the highly localized, sharply curved buckles observed in our experiments rather than a single gentle arc. This picture is further supported by inspection of the most unstable eigenmodes predicted by the theory: Just above the critical buckling length, $\Lambda^{*}$, the most unstable mode is a single long-wavelength deformation, while at larger Λ, the most unstable mode becomes increasingly oscillatory and spatially localized near the chain center, where the compressive viscous load is largest (Fig. 5 *C* and *D* and *SI Appendix*).

The transition predicted by the linear theory mirrors our experimental observation that the lowest-n chains (e.g., those pinned) exhibit long-wavelength arcs reminiscent of k=1 deformations, while less-constrained chains develop multiple sharply curved buckles with n>1. Some experimental realizations, like that shown in Fig. 3, exhibit finite-amplitude, sharply curved buckles reminiscent of the wrinkling-to-folding transition in elastic media under large compressive loads (81–83). The linear theory presented here does not capture these large-amplitude postbuckling shapes, and a fully nonlinear treatment of the LC director field and the associated surface tractions will be needed to describe them in future work.

## Discussion

By combining experiments and continuum mechanical theory, we have shown that liquid crystalline environments can shape bacterial colony morphology through purely physical interactions. Nonmotile bacteria proliferating in a nematic LC form long, serpentine, single-cell-wide chains aligned along the director—a morphology that arises because LC elasticity keeps proliferating cells oriented end-to-end. As these chains elongate, growth-induced viscous stresses accumulate along their contours, eventually overcoming the elastic penalty imposed by the surrounding nematic and triggering highly localized buckling that starkly contrasts to the long-wavelength bending typical of classical Euler buckling—conceptually similar to how the elastic cytoskeleton suppresses bending of microtubules inside cells (84).

Our theoretical analysis provides quantitative principles that describe the dependence of chain buckling on the Ericksen number and LC–cell surface anchoring strength, motivating future studies that explore variations in these parameters further. Indeed, while our theory quantitatively captures the decrease in critical buckling length with increasing LC concentration, the spread in measured $L^{*}$ values suggests additional variability—likely from cell-to-cell differences in anchoring strength, and possibly from imperfection sensitivity or subcritical effects that could be disentangled through analysis of a fully nonlinear model. In addition, future extensions of our theory could incorporate: i) *Discrete representation of cells.* Our continuum model treats each chain as a smooth filament. Because the most unstable wavelengths predicted by the theory (∼50 to 200μm) are much larger than a single cell (∼2μm), we expect this approximation to introduce small errors near the onset of instability. Moreover, the continuum-modeled chains have no intrinsic bending stiffness; instead, the resistance to bending arises entirely from the LC elastic coupling between adjacent cells. Steric or adhesive cell–cell interactions could provide an additional contribution. ii) *Confining boundaries.* Surface effects enter only through proximity-dependent resistive coefficients; a full slender-body treatment in the presence of a surface would refine the quantitative predictions. iii) *Nonuniform growth.* Our growth law assumes spatially uniform elongation along the chain. Variability in growth rates within a chain could shift the local compression profile. iv) *Elastic anisotropy of DSCG.* Our theory uses the one-constant approximation. Published measurements indicate that the splay ($K_{1}$) and bend ($K_{3}$) constants of DSCG are comparable in magnitude (both ∼10pN, with $K_{1}/K_{3}$ depending on concentration and temperature), while the twist constant $K_{2}$ is an order of magnitude smaller (9). A more general treatment of anisotropy may shed additional light on the large variance observed in the buckling lengths. Future experiments could also provide a more stringent test of the theoretical predictions by exploring LC concentrations over a broader range, as well as varying the bacterial growth rate and cellular confinement.

Intriguingly, bacterial colony morphologies reminiscent of the chains studied here have been observed in various biological contexts. However, their origin is typically attributed to specific adhesive interactions holding cells together (85–88). Here, we reveal a fundamentally distinct mechanism by which proliferating bacteria can form such long, single-cell-wide chains: Through mechanical interactions with a surrounding liquid crystal, requiring neither specific cell–cell adhesins nor dedicated genetic programs. This finding, which complements our previous study of “cables” formed by cells proliferating in polymer solutions (89), highlights how purely physical interactions with surrounding complex fluids—such as host mucus (1–3), extracellular polymeric substances in biofilms (4), and dense suspensions of filamentous phages (5–7)—can profoundly shape growing bacterial colonies.

What are the biological implications of chain formation? Our findings suggest that LC-mediated chain formation could influence how nonmotile bacteria explore space, access nutrients, and interact with external stressors. Long chains have large surface-area-to-volume ratios, potentially altering susceptibility to antibiotics or phages (5–7, 49–52); alternatively, the extended chain morphology could enable colonies to reach new environmental niches (86, 90) or nutrient sources (47, 48). The buckling instability itself may serve as a mechanical signal that triggers phenotypic changes in the cells themselves; indeed, our theory suggests that the compressive force in chains reaches ∼1pN at the onset of buckling—comparable to the forces that drive bacterial mechanosensitive responses (91), such as biofilm formation. Investigating these possibilities, both in quiescent conditions and under flow (92, 93), will be an important direction for future work.

Our work also opens directions in soft matter physics. While colloidal assembly in LCs has been extensively studied (55, 68, 94, 95), our results highlight that qualitatively new behaviors emerge when the constituent particles can proliferate (26): Unlike passive systems, in our system the particle number, particle position along the chain, and compressive load along the chain all evolve in time due to growth. Moreover, the growth-induced viscous load is distributed along the chain in a manner distinct from the end-applied loads of classical Euler buckling and from the body-force-driven compression of sedimenting filaments. Each bacterial chain itself has no intrinsic bending stiffness—cells are unattached and could in principle freely rotate relative to one another. Instead, the LC elasticity supplies an effective bending rigidity that nonlocally couples chain deformations to the surrounding director field; this nonlocal coupling, combined with the distributed compressive load, promotes instability at shorter wavelengths than classical Euler buckling. This interplay between growth-generated stresses, LC elasticity, and surface anchoring creates a rich design space for controlling colony morphology, and potentially for engineering living materials with prescribed architectures. Extending our framework to other anisotropic environments, such as cholesteric LCs or nematic gels with tunable elasticity, could reveal additional morphogenetic programs accessible through physical interactions alone (96).

## Materials and Methods

### Preparation of Cells.

#### E. coli.

We incubated an overnight culture of *E. coli* strain W3110 in 2 w/w% Lennox Lysogeny Broth (LB) at 30 °C. Next, we inoculated 20 μL from the overnight culture in 2 mL of LB for 3 h such that, at the time of imaging, bacteria would reach mid-exponential phase. The *E. coli* constitutively express green fluorescent protein (GFP) throughout their cytoplasm and have a deletion of the flagellar regulatory gene *flhDC* that renders them nonmotile.

#### P. aeruginosa.

We incubated an overnight culture of *P. aeruginosa* strain PA01 in LB, supplemented with carbenicillin (200 μg/mL) to preserve the strain’s fluorescence, at 37 °C. Next, we inoculated 20 μL from the overnight culture in 2 mL of LB for 3 h. We used a strain with a double *fliC* and *pilA* deletion that renders the cells nonmotile, and verified that these cells are nonmotile using direct visualization.

#### V. cholerae.

We incubated an overnight culture of *V. cholerae* 01 biovar El Tor strain C6706 in LB at 37 °C. Next, we inoculated 20 μL from the overnight culture in 2 mL of LB for 3 h at 37 °C. We used a strain with several gene deletions: It has a deletion of *pomA*, which renders the cells nonmotile, and we verified that these cells are nonmotile using direct visualization. It also has deletions of *rbmA*, *bap1*, *rbmC*, and *vpsL*, which renders the cells as nonbiofilm formers.

### Preparation of Liquid Crystal Solution.

We purchased liquid crystal disodium chromoglycate (DSCG) from Thermo Fisher Scientific. DSCG stock solutions were prepared in LB and put in a spinning rotor until full dissolution was observed. The DSCG solution was then filtered using a 5 μm membrane from GE Healthcare life sciences. To obtain liquid crystal solutions at the desired concentrations we diluted the stock solutions with the same solvent.

### Imaging of Bacteria in Liquid Crystal Solution.

#### E. coli in liquid crystal.

To image fluorescent *E. coli* we used custom built rectangular PDMS devices with dimensions 2 mm in width, 22 mm in length, and 25 μm in height. The device was filled with the liquid crystal solution containing a well-mixed bacterial population at a concentration $c_{\text{cell}}\;\approx 10^{5}$ to $10^{6}$ cells/mL. Next, we sealed inlet and outlet ports of the PDMS channel with paraffin oil to minimize evaporation. Finally, we imaged the dish with a 20× magnification air objective (Fig. 1*A*–*C*) or 60× magnification oil objective with added 1.5× zoom (Fig. 1*F*) in a Nikon AXR inverted laser-scanning confocal microscope with the stage maintained at $30\pm \;1^{{}^{\circ}}$C. We recorded images throughout the sample every 1 to 10 min over ∼6 to 14 h. To obtain the images in Fig. 1 *C* and *F*, we inserted a linear polarizer on the lightpath toward the transmittance detector.

To investigate the reversibility of chain formation (Fig. 2*B*), we used a transparent-walled glass-bottom petri dish sealed with an overlying PDMS slab. We additionally pierced the PDMS slab with three 20-gauge needles, sealed using optical glue, and connected to a Harvard Apparatus 11 Elite syringe pump to provide fluid in/outflow. In particular, one needle acts as an inlet for injection of the test solution containing ≈4 × 10^5^ cells/mL suspended in 15 w/w% DSCG solution; the second acts as an inlet for injection of DSCG-free LB media; and the third acts as the outlet. We then imaged the sealed chamber from below using a Nikon A1R+ inverted laser scanning confocal microscope with the stage maintained at $30\pm \;1^{{}^{\circ}}$C as before, first under quiescent no-flow conditions with the chamber filled with the test solution containing cells and LC. Once chains have formed, we then pump LC-free fluid through at a flow rate of 2.5 μL/min to remove LC-containing solution.

To determine the onset of chain buckling (Fig. 3), we i) smooth the confocal micrographs of growing chains using a 2D Gaussian filter, ii) binarize the images using Otsu’s method of thresholding (97), iii) skeletonize the individual chains using the medial axis transform and thereby determine their backbones, and iv) directly measure the tortuosity of each identified chain backbone. To characterize postbuckling shapes, we compute the unbiased spatial autocorrelation function of the normalized local curvature vector along each skeletonized backbone and its power spectrum, from which we extract the dominant wavenumber $q_{\text{c}}$, the correlation length $\xi_{c}=2\pi /q_{\text{c}}$, and the correlation number $n=L/\xi_{c}$; details are provided in *SI Appendix*.

#### P. aeruginosa and V. cholerae in liquid crystal.

We filled individual wells of a glass-bottom 96-well plate with 100 μL of the solution and 0.1μL of the inoculum of cells such that their initial concentration is ≈10^6^ cells/mL. We stored the plate in a static 30°C incubator and imaged the plate at different time points: For *P. aeruginosa*, we imaged at 120 and 840 min after the start of incubation, and for *V. cholerae*, we imaged at 180 and 360 min after incubation as shown in *SI Appendix*, Fig. S5 *A* and *B*. For all images taken, we used a 20× air objective mounted in a Nikon A1R+ inverted laser scanning confocal microscope with the stage maintained at room temperature.

## Supplementary Material

Appendix 01 (PDF)

Movie S1. Non-motile *E. coli* proliferating in LB media without DSCG.

Movie S2. Non-motile *E. coli* proliferating in LB media supplemented with 15 w/w% DSCG.

Movie S3. Non-motile *E. coli* proliferating in LB media supplemented with 15 w/w% DSCG. At *t* = 0 min we flush a suspension of chains with LC free liquid at a flow rate of 2.5 μL/min, thus decreasing the LC concentration as time increases.

Movie S4. Non-motile *E. coli* proliferating in LB media supplemented with 15 w/w% DSCG. At *t* = 0 min we flush a suspension of chains with 15 w/w% DSCG solution at a flow rate of 2.5 μL/min keeping the LC concentration constant as time increases.

## Data Availability

All data are available on Zenodo (https://doi.org/10.5281/zenodo.17917861) (98). All other data are included in the manuscript and/or supporting information.
