# Supplementary material for: Morphogenesis of bacterial colonies in liquid crystalline environments
Source: Proc Natl Acad Sci U S A. 2026 Aug 18;123(34):e2536343123. doi: 10.1073/pnas.2536343123 (PMC13506060; doi:10.1073/pnas.2536343123)
Supplement: Supplementary file 1 [file pnas.2536343123.sapp.pdf]

PNAS

Supporting Information for

Morphogenesis of bacterial colonies in liquid crystalline environments

Sebastian Gonzalez La Corte, Thomas G. J. Chandler, Saverio E. Spagnolie, Ned S. Wingreen, and Sujit S. Datta

Correspondence to Sujit S. Datta.
E-mail: ssdatta@caltech.edu

This PDF file includes:

Supporting text
Figs. S1 to S12
Table S1
Legends for Movies S1 to S4
SI References

Other supporting materials for this manuscript include the following:

Movies S1 to S4

Supporting Information Text

Osmotic pressure of nematic liquid crystal solutions

To estimate the osmotic pressure of nematic liquid crystals (LCs) we use the expression (1)

$$\Pi_{\mathrm{osm}} = 3 n_{\mathrm{p}} k_{\mathrm{B}} T, \tag{1}$$

where n_{p} is the number density of nematic liquid crystal molecules, k_{B} is Boltzmann's constant, and T is the absolute temperature. We estimate the number density as $n_{\mathrm{p}} \approx 4\phi/(l^2 D\pi)$ (1, 2), where ϕ is the fractional volume occupied by the LC particles, D is the average diameter of the LC particles, and l is the average length of the LC particles. Inputting the values of Table S1 renders $\Pi_{\mathrm{osm}} = 2000\,\mathrm{Pa}$.

Quantitative characterization of buckled chain shapes

A defining feature of the buckled chains is that their high-curvature regions are spatially localized, occupying only a finite portion of the total arclength rather than distributing uniformly along the contour [main text Fig. 3(a)]. This behavior contrasts starkly with the classical Euler buckling of a slender filament under an end-applied load, for which the unstable mode spans the entire contour. To quantify this localization and track its temporal evolution, we analyze the spatial statistics of the chain centerline following the onset of buckling.

For each chain at each time point after buckling onset, we first compute the unbiased spatial autocorrelation function (ACF) of the normalized local curvature vector $\boldsymbol{\kappa}(s,t)$ along the contour,

$$\mathrm{ACF}(\Delta s, t) = \frac{1}{s_{\max} - \Delta s} \int_0^{s_{\max} - \Delta s} \boldsymbol{\kappa}(s,t) \cdot \boldsymbol{\kappa}(s + \Delta s, t)\, \mathrm{d}s, \tag{2}$$

where s is the arclength along the centerline, t is the time, and Δs is the arclength lag. To avoid limited-data edge artifacts at long lags, we restrict the integration domain $s_{\max}$ to 75–90% of the total contour length on a chain-by-chain basis. The ACF decays rapidly at small lags, reflecting the loss of directional correlation between curvature vectors over short distances. It then exhibits distinct local extrema at intermediate lags [main text Fig. 3(d), inset]: because the localized, high-curvature deformation zones act as repeating spatial features, the alignment between curvature vectors is partially recovered when the lag matches the characteristic spacing between these zones, generating a local maximum in the ACF.

To extract the characteristic spatial scale of this periodicity, we compute the power spectrum of each ACF profile,

$$P(q,t) = \left| \frac{1}{s_{\max}} \int_0^{s_{\max}} \left[\mathrm{ACF}(\Delta s, t) - \overline{\mathrm{ACF}}(t) \right] e^{-iq\Delta s}\, \mathrm{d}\Delta s \right|^2, \tag{3}$$

where q is the wavenumber conjugate to the arclength lag and $\overline{\mathrm{ACF}}(t)$ is the mean of the ACF profile at time t. Although the power is distributed across several wavenumbers, a distinct peak emerges at a dominant wavenumber $q_{\mathrm{c}}(t)$ [main text Fig. 3(d)], from which we define the characteristic correlation length $\xi_{\mathrm{c}}(t) \equiv 2\pi/q_{\mathrm{c}}(t)$.

Tracking both the total contour length L and the correlation length ξ_{c} as the chain elongates, we find that although each grows individually, their ratio—the correlation number $n \equiv L/\xi_{\mathrm{c}}$—saturates at a constant value of ≈ 2.4 for the representative chain shown in main text Fig. 3(e). This saturation indicates that the chain maintains a fixed number of correlation intervals within its contour as it lengthens. The correlation number provides a compact metric for the buckling morphology: $n \approx 1$ corresponds to a single arc spanning the chain, as in classical Euler buckling, whereas $n > 1$ is the quantitative signature of localized buckling, in which the curvature periodicity is shorter than the contour length. We measure $n > 1$ for the vast majority of buckled chains in our dataset [main text Fig. 3(f)]. Larger values of n correspond to high-curvature sectors confined to progressively smaller fractions of the arclength.

This localized-buckling motif is robust across LC concentrations and bacterial species. Applying the same analysis to nonmotile *Pseudomonas aeruginosa* and *Vibrio cholerae* proliferating in 18 w/w% DSCG yields correlation numbers $n > 1$ in every case [Figs. S9–S12], confirming that the phenomenon does not depend on the specific cell type. We attribute the chain-to-chain variability in n to differences in local confinement and boundary conditions, since the chains buckle in close proximity to the surfaces of the imaging device [Fig. S6]; consistent with this picture, chains with both endpoints firmly pinned exhibit a correlation number $n = 1$, corresponding to the long-wavelength limit [Fig. S8].

Estimation of chain distance from a surface

We observe that buckling occurs in chains that have at least one endpoint transiently pinned, suggesting that bacterial chains come into contact with the surfaces of the imaging device (the chain's endpoints may come into contact with the glass bottom surface (3) or with the top PDMS surface (4)). Three-dimensional confocal reconstructions confirm this picture directly: growing chains lie close to, and elongate parallel to, the top and bottom walls of the device, and buckle in their immediate vicinity [Fig. S6]. To incorporate these surface effects into the theoretical calculations, we assume that the filament is growing a distance, h, from a surface (Fig. S3). To estimate h experimentally, we track the z-coordinate of a chain's endpoints throughout the experiment. Next, we calculate the standard deviation of the z-coordinate time series. The standard deviation yields an

estimate of the chain's fluctuations near the surface of the imaging device, providing an estimate of a chain's average distance from the surface. We repeated this procedure for multiple buckling chains. The averages of these measurements are shown in Table S1 for different DSCG concentrations.

Buckled chains remain planar and parallel to the confining walls

The geometry of confinement bears directly on two assumptions of the continuum model: that the in-plane drag on the chain is isotropic (i.e., the resistance tensor has a single transverse coefficient $\zeta_\perp$), and that wall interactions can be captured through the boundary conditions on the chain endpoints rather than along its full length. Analyzing the three-dimensional centerline configurations extracted from confocal reconstructions of buckling chains in 18 w/w% DSCG [Fig. S6] confirms these assumptions.

In particular, throughout growth and buckling, each chain remains close to and elongates parallel to the top or bottom wall, with its z-extent confined to within $\approx 1\,\mu$m of the wall—comparable to the cell radius a and far smaller than the chain length L [lower panels of Fig. S6(a)–(c)]. Second, the buckling deformation develops predominantly in the plane parallel to the wall (the xy-plane), rather than out of it. We quantify these observations across our full dataset using two metrics [Fig. S7]: The chain tilt angle—the angle between the chain's principal axis and the xy-plane—has a mean near zero in both LC concentrations [0.1° and −0.2° for 15 and 18 w/w%, respectively; Fig. S7(a)], confirming that chains grow horizontally. The chain aspect ratio—the ratio of the in-plane (xy) to out-of-plane (xz) extent of the buckled shape—has a mean $\approx$ 8–9 [Fig. S7(b)], confirming that the buckled conformation is largely contained in the xy-plane.

These observations justify the modeling choices in two ways. Because the buckling is planar, the relevant transverse drag is that for motion within the xy-plane, so a single transverse coefficient $\zeta_\perp$ suffices and the in-plane isotropy assumption does not affect the predicted onset of buckling. The proximity to the wall, $h \sim a \ll L$, is precisely the regime in which the wall-corrected resistive-force coefficients $\zeta_\parallel \approx 2\pi\mu/\log(2h/a)$ and $\zeta_\perp \approx 2\zeta_\parallel$ apply (5), which we adopt in the model. A chain pinned to a wall and elongating parallel to it does experience wall interactions along its contour, not only at its endpoints; treating the pinning as an endpoint condition is therefore an idealization. As we show below, however, the two limiting endpoint conditions we consider (free-free and fixed-free) bound the observed behavior and yield critical buckling lengths that differ only by a factor of ≈ 1.5 [Fig. S1], so this idealization does not qualitatively affect our conclusions. A full slender-body treatment in the presence of a wall would refine the quantitative predictions but likely does not alter them qualitatively.

Mathematical modeling

Slender filament growth and kinematics. The bacterial chain will be treated as a continuum filament of length $L(t)$, which starts at $t = 0$ in a straight configuration with centerline $\boldsymbol{X}_0(S)$ parameterized by the arclength $S \in [-L(0)/2, L(0)/2]$ (the reference frame). At time t, the centerline position is written as $\boldsymbol{x}_0(s(S,t),t)$, where $s \in [-L(t)/2, L(t)/2]$ is the current arclength parameter and $|\partial_s \boldsymbol{x}_0| = 1$.

Assuming a uniform growth law for each unit of bacterial length, we have $\partial s/\partial S = \alpha$ and $\dot{s} \equiv \partial_t s(S,t) = \alpha s(S,t)$. Hence, with $s(S,0) = S$, we have naturally that $s(S,t) = S\exp(\alpha t)$.

The material velocity, denoted by $\boldsymbol{V}(S,t)$, of the filament centerline is given by the time rate of change of the current position for fixed material coordinate S:

$$\boldsymbol{V}(S,t) = \frac{\mathrm{d}}{\mathrm{d}t}\boldsymbol{x}_0(s(S,t),t)\Big|_S = \partial_t \boldsymbol{x}_0\Big|_s + \dot{s}\,\partial_s \boldsymbol{x}_0 =: \boldsymbol{v}(s(S,t),t) + \alpha s(S,t)\hat{\boldsymbol{s}}(s(S,t),t), \qquad [4]$$

where $\hat{\boldsymbol{s}} \equiv \partial_s \boldsymbol{x}_0(s(S,t),t)$ is the unit tangent vector along the centerline. We recognize the final two terms as the rigid body motion component and the growth component, respectively. We further decompose the rigid body motion into its tangential and normal parts. At time t,

$$\boldsymbol{v}(s,t) = v_\parallel(s,t)\hat{\boldsymbol{s}}(s,t) + \boldsymbol{v}_\perp(s,t), \qquad [5]$$

where $\hat{\boldsymbol{s}} \cdot \boldsymbol{v}_\perp = 0$. Continuity demands that $|\partial_s \boldsymbol{x}_0| = 1$ at all times, which implies that

$$0 = \frac{1}{2}\frac{\mathrm{d}}{\mathrm{d}t}|\partial_s \boldsymbol{x}_0|^2 = \partial_s \boldsymbol{x}_0 \cdot \partial_{st}\boldsymbol{x}_0 = \hat{\boldsymbol{s}} \cdot \partial_s(\partial_t \boldsymbol{x}_0) = \hat{\boldsymbol{s}} \cdot \partial_s \boldsymbol{v}. \qquad [6]$$

Filament geometry. For a small planar displacement of the filament, u, we write the centerline position as

$$\boldsymbol{x}_0(s,t) = s\hat{\boldsymbol{x}} + u(s,t)\hat{\boldsymbol{y}} + \mathcal{O}(Lu_s^2), \qquad [7]$$

where $\{\hat{\boldsymbol{x}}, \hat{\boldsymbol{y}}, \hat{\boldsymbol{z}}\}$ denote the unit Cartesian vectors, with $\hat{\boldsymbol{x}}$ pointing along the length of the undeformed filament. Connecting this definition to Eq. 4, we must then have that $\boldsymbol{v}(s,t) = (\partial u/\partial t)\hat{\boldsymbol{y}} + \mathcal{O}(Lu_s^2)$. To study the initial instability we will assume that $|u_s| \ll 1$. The centerline unit tangent vector, $\hat{\boldsymbol{s}} = \partial_s \boldsymbol{x}_0$, is accompanied by normal and binormal unit vectors along the centerline, $\{\hat{\boldsymbol{n}}, \hat{\boldsymbol{z}}\}$, where

$$\hat{\boldsymbol{s}} = \hat{\boldsymbol{x}} + u_s\hat{\boldsymbol{y}} + \mathcal{O}(u_s^2), \qquad [8a]$$

$$\hat{\boldsymbol{n}} = -u_s\hat{\boldsymbol{x}} + \hat{\boldsymbol{y}} + \mathcal{O}(u_s^2), \qquad [8b]$$

forming an orthonormal triad. Continuity, via Eq. 6, then gives that $\partial_s v_\parallel(s,t) = O(u_s^2)$. At leading order, the rigid body component of the filament velocity must be constant in the tangential direction, $v_\parallel(s,t) = v_\parallel(t)$, or else the filament would rupture.

Each cross-section of the filament is assumed to be circular with diameter $2a$. The filament surface, denoted by ∂D (neglecting the filament ends), is then parameterized by $(s,\eta) \in [-L(t)/2, L(t)/2] \times [0, 2\pi)$, as

$$\boldsymbol{x}(s,\eta) = \boldsymbol{x}_0(s) + a\hat{\boldsymbol{e}}_\perp(s,\eta), \tag{9}$$

where $\hat{\boldsymbol{e}}_\perp(s,\eta) = \cos\eta\,\hat{\boldsymbol{n}}(s) + \sin\eta\,\hat{\boldsymbol{z}}$. At each point on the surface, $\boldsymbol{x}(s,\eta) \in \partial D$, we have another orthonormal triad $\{\hat{\boldsymbol{\xi}}, \hat{\boldsymbol{\zeta}}, \hat{\boldsymbol{\nu}}\}$, composed of two unit tangent vectors on the surface, $\hat{\boldsymbol{\xi}} = \boldsymbol{x}_s/|\boldsymbol{x}_s|$ and $\hat{\boldsymbol{\zeta}} = \boldsymbol{x}_\eta/|\boldsymbol{x}_\eta|$, and the unit normal vector, which points into the LC, $\hat{\boldsymbol{\nu}} = \hat{\boldsymbol{\zeta}} \times \hat{\boldsymbol{\xi}}/|\hat{\boldsymbol{\zeta}} \times \hat{\boldsymbol{\xi}}|$. With the centerline displacement assumed to be small, these are given by

$$\boldsymbol{x}(s,\eta) = s\hat{\boldsymbol{x}} + a\hat{\boldsymbol{r}} + \mathcal{O}(Lu_s), \tag{10a}$$
$$\hat{\boldsymbol{\zeta}}(s,\eta) = -\sin\eta\hat{\boldsymbol{n}}(s) + \cos\eta\hat{\boldsymbol{z}} + \mathcal{O}(u_s^2), \tag{10b}$$
$$\hat{\boldsymbol{\xi}}(s,\eta) = \hat{\boldsymbol{s}}(s) + \mathcal{O}(u_s^2), \tag{10c}$$
$$\hat{\boldsymbol{\nu}}(s,\eta) = \cos\eta\hat{\boldsymbol{n}}(s) + \sin\eta\hat{\boldsymbol{z}} + \mathcal{O}(u_s^2), \tag{10d}$$

where $\hat{\boldsymbol{r}}(\eta) = \cos\eta\hat{\boldsymbol{y}} + \sin\eta\hat{\boldsymbol{z}}$ and $\hat{\boldsymbol{\eta}}(\eta) = -\sin\eta\hat{\boldsymbol{y}} + \cos\eta\hat{\boldsymbol{z}}$ are the radial and azimuthal coordinate vectors, respectively. Later we will use the fact that $\hat{\boldsymbol{\zeta}} = \hat{\boldsymbol{\eta}} + \mathcal{O}(u_s)$, $\hat{\boldsymbol{\nu}} = \hat{\boldsymbol{r}} + \mathcal{O}(u_s)$, and $\hat{\boldsymbol{s}} = \hat{\boldsymbol{x}} + \mathcal{O}(u_s)$, i.e. recovering cylindrical coordinates at leading-order.

Force balance and filament dynamics. Force balance is considered at each segment along the filament. At a station s, in the limit of large viscous damping relative to the inertial effects (i.e., Stokes flow), the resultant force (per unit length) acting on the filament is zero at all times. We decompose this force as

$$\boldsymbol{f}(s,t) = \boldsymbol{f}^{\text{viscous}}(s,t) + \partial_s\boldsymbol{F}(s,t) + \boldsymbol{f}^{\text{LC}}(s,t) = \boldsymbol{0}, \tag{11}$$

where $\boldsymbol{f}^{\text{viscous}}$ is the viscous drag, $\partial_s\boldsymbol{F}$ is the differential elastic force, and $\boldsymbol{f}^{\text{LC}}$ is the LC restoring force. We shall compute each of these terms in turn.

First is the viscous drag, which we model using a resistive force approximation,

$$\boldsymbol{f}^{\text{viscous}} = -\boldsymbol{\mathcal{R}} \cdot \boldsymbol{V}, \tag{12}$$

where $\boldsymbol{\mathcal{R}}$ is a resistance tensor,

$$\boldsymbol{\mathcal{R}} = \zeta_\parallel \hat{\boldsymbol{s}}\hat{\boldsymbol{s}} + \zeta_\perp(\mathbf{I} - \hat{\boldsymbol{s}}\hat{\boldsymbol{s}}). \tag{13}$$

The drag coefficients $\zeta_\parallel$ and $\zeta_\perp$ penalize motion in the filament tangent and normal directions, respectively. In terms of the rigid body motion and growth components of the filament velocity, we may write

$$\boldsymbol{f}^{\text{viscous}} = -\left[\zeta_\parallel \hat{\boldsymbol{s}}\hat{\boldsymbol{s}} + \zeta_\perp\left(\mathbf{I} - \hat{\boldsymbol{s}}\hat{\boldsymbol{s}}\right)\right] \cdot (\boldsymbol{v} + \alpha s\hat{\boldsymbol{s}}) = -\zeta_\parallel(v_\parallel + \alpha s)\hat{\boldsymbol{s}} - \zeta_\perp \boldsymbol{v}_\perp. \tag{14}$$

For reference, in a Newtonian fluid with viscosity μ, we would have $\zeta_\parallel \approx 2\pi\mu/\log(L/2a)$ and $\zeta_\perp \approx 2\zeta_\parallel$ at leading-order in $1/\log(L/2a)$, assumed small; or, we instead have $\zeta_\parallel \approx 2\pi\mu/\log(2h/a)$ and $\zeta_\perp \approx 2\zeta_\parallel$ if the filament is moving a distance $h \ll L$ away from a wall (5).

Next is the differential elastic force due to the connectivity of the filament itself. We assume that the filament cannot support a bending moment; hence, from the angular momentum balance, we can write the force density as a tangential piece alone, $\boldsymbol{F}(s,t) = T(s,t)\hat{\boldsymbol{s}}$. With this, we can decompose the force balance, Eq. 11, into its tangential and normal components,

$$-\zeta_\parallel(v_\parallel + \alpha s) + \partial_s T = 0, \tag{15a}$$
$$-\zeta_\perp \boldsymbol{v}_\perp + (\mathbf{I} - \hat{\boldsymbol{s}}\hat{\boldsymbol{s}}) \cdot \left(\boldsymbol{f}^{\text{LC}} + T\partial_s\hat{\boldsymbol{s}}\right) = \boldsymbol{0}, \tag{15b}$$

respectively, where we have used that $\hat{\boldsymbol{s}} \cdot \boldsymbol{f}^{\text{LC}} = 0$ (see the subsection on LC director field and traction below). If both ends of the filament are free, the end tensions must equate, $T(\pm L/2) = T_L = \mathcal{O}(w^2)$, with $w = aW/K$ the dimensionless anchoring strength. Integrating the tangential force balance, Eq. 15a, thus yields $v_\parallel = 0$, as well as an expression for the tension,

$$T(s,t) = T_L - \frac{\zeta_\parallel \alpha}{2}\left(\frac{L(t)^2}{4} - s^2\right). \tag{16}$$

This baseline tension can be negative (compression), which has the potential to buckle the filament. This compression can be even more pronounced if one end of the filament meets additional resistance, due to surface interactions, for instance.

Consider the extreme case when one endpoint (e.g., the one at $s = -L/2$) is pinned (hinged), which requires an additional tangential force F there. With $\boldsymbol{V}(-L/2,t) = [v_\parallel(t) - \alpha L(t)/2]\hat{\boldsymbol{x}} + \boldsymbol{v}^\perp(-L/2,t) = \boldsymbol{0}$, we find that $v_\parallel(t) = \alpha L/2$ and $\boldsymbol{v}^\perp(-L/2,t) = \boldsymbol{0}$. Integrating Eq. (15a), we find that $-\zeta_\parallel \alpha L^2/2 + T_L - T_{-L} = 0$ for $T_{\pm L} \equiv T(\pm L/2)$. Using the endpoint force

balance, $F + T_{-L} = 0$, we find that the required tangential force is $T_{-L} = -F = T_L - \zeta_\parallel \alpha L^2/4$. In this pinned problem the baseline tension is given instead by

$$T(s,t) = T_L - \frac{\alpha\zeta_\parallel}{2}\left(\frac{L(t)}{2} - s\right)\left(\frac{3L(t)}{2} + s\right). \tag{17}$$

For the free filament, from Eq. (16), the tension is smallest (and becomes negative, as a compression) at the midpoint, $s = 0$. For the pinned filament, from Eq. (17) the tension is smallest at the point of pinning, $s = -L/2$, and it can be up to 4 times larger in the absence of T_L (i.e. $-\alpha\zeta L^2/8$ vs. $-\alpha\zeta L^2/2$).

In either case, using $\boldsymbol{v}_\perp = u_t\hat{\boldsymbol{y}}[1 + \mathcal{O}(u_s)]$, Eq. 15b may be written as

$$\zeta_\perp u_t = T(s)u_{ss} + \hat{\boldsymbol{y}} \cdot \boldsymbol{f}^{\mathrm{LC}}(s), \tag{18}$$

where $T(s)$ is given in Eq. 16, for free-free ends, or Eq. 17, for fixed-free ends. In the free-free case, the boundary conditions are selected to match the LC director field far away (assumed to be the $\hat{\boldsymbol{x}}$-direction), i.e., $u_s(\pm L/2) = 0$. In the fixed-free case, the boundary conditions instead take the form $u(-L/2) = u_s(L/2) = 0$. Although Eq. 18 appears straightforward, the time-dependence in $L(t)$ presents an additional challenge, which is addressed below by a change of variables to the reference frame.

LC director field and traction. Before we can determine the traction on the filament due to the surrounding LC environment, we need to determine the LC configuration. Fortunately, the system is evolving slowly, so we can determine the LC field in a quasi-static manner, i.e., solving an equilibrium problem for each filament configuration. Here, we let $L = L(t)$ and drop all arguments of time since the LC director field is determined instantaneously at any given time t.

Far from the bacterial chain, the LC director field is assumed to be uniform in the $\hat{\boldsymbol{x}}$ direction. Elsewhere, at a position $\boldsymbol{x}$ in the fluid, the LC director field is represented as $\boldsymbol{n}(\boldsymbol{x}) = \hat{\boldsymbol{x}} + \boldsymbol{p}(\boldsymbol{x}) + \mathcal{O}(u_s^2)$ with $\boldsymbol{p} \cdot \hat{\boldsymbol{x}} = \boldsymbol{0}$, where $\boldsymbol{p} = \mathcal{O}(u_s)$ in order to have a balance between the deflection and the director field. With this approximation, the Ericksen torque stress tensor and projected molecular field are given by

$$\boldsymbol{\Pi} = K\nabla\boldsymbol{n} = K\nabla\boldsymbol{p}\left[1 + \mathcal{O}(u_s)\right], \tag{19a}$$
$$\boldsymbol{h} = \mathbf{P}(\boldsymbol{n}) \cdot (\nabla \cdot \boldsymbol{\Pi}) = K\nabla^2\boldsymbol{p}\left[1 + \mathcal{O}(u_s)\right], \tag{19b}$$

respectively, for the single (Frank) elastic constant K and projection operator $\mathbf{P}(\boldsymbol{n}) \equiv \mathbf{I} - \boldsymbol{n}\boldsymbol{n}$, see Ref. (6). At equilibrium, $\boldsymbol{h} = \boldsymbol{0}$ and so $\nabla^2\boldsymbol{p} = \boldsymbol{0}$ with $\boldsymbol{p} \to \boldsymbol{0}$ as $|\boldsymbol{x}| \to \infty$. On the boundary, $\boldsymbol{x} \in \partial D$, we have the projected surface molecular field

$$\boldsymbol{h}_{\mathrm{surf}} = \mathbf{P}(\boldsymbol{n}) \cdot \left[\hat{\boldsymbol{\nu}} \cdot \boldsymbol{\Pi} - \frac{\partial \mathcal{F}_{\mathrm{surf}}}{\partial \boldsymbol{n}}\right] = \left[K\partial_\nu \boldsymbol{p} - \mathbf{P}(\boldsymbol{n}) \cdot \frac{\partial \mathcal{F}_{\mathrm{surf}}}{\partial \boldsymbol{n}}\right]\left[1 + \mathcal{O}(u_s)\right], \tag{20}$$

where $\mathcal{F}_{\mathrm{surf}}$ is a surface energy that describes how the director field is anchored to the bacteria (to be defined), see Ref. (7). At equilibrium, $\boldsymbol{h}_{\mathrm{surf}}(\boldsymbol{x}) = \boldsymbol{0}$ for $\boldsymbol{x} \in \partial D$. Note from Eq. 10a that $\boldsymbol{h}_{\mathrm{surf}}(\boldsymbol{x}) = \boldsymbol{h}_{\mathrm{surf}}(s\hat{\boldsymbol{x}} + a\hat{\boldsymbol{r}}) + \mathcal{O}(u\nabla\boldsymbol{h}_s)$ for $\boldsymbol{x} \in \partial D$, so ∂D is effectively a cylinder of radius a aligned with $\hat{\boldsymbol{x}}$. We thus introduce a cylindrical polar coordinate system $(s, r, \eta) \in [-L/2, L/2] \times [a, \infty) \times [0, 2\pi)$.

Surface anchoring and director field. On the filament surface, the LC is assumed to satisfy weak (i.e. finite strength) degenerate tangential anchoring conditions, with surface energy

$$\mathcal{F}_{\mathrm{surf}} = \frac{W}{2}(\boldsymbol{n} \cdot \hat{\boldsymbol{\nu}})^2, \tag{21}$$

for the unit normal vector $\hat{\boldsymbol{\nu}} = \hat{\boldsymbol{r}} - u_s\cos\eta\hat{\boldsymbol{x}} + \mathcal{O}(u_s^2)$ and anchoring strength W, see Ref. (8). Since $\boldsymbol{n} \cdot \hat{\boldsymbol{\nu}} = \boldsymbol{p} \cdot \hat{\boldsymbol{r}} - u_s\cos\eta + \mathcal{O}(u_s^2)$, the surface molecular field is

$$\boldsymbol{h}_{\mathrm{surf}} = \left[K\partial_r\boldsymbol{p} - W(\boldsymbol{p} \cdot \hat{\boldsymbol{r}} - u_s\cos\eta)\hat{\boldsymbol{r}}\right]\left[1 + \mathcal{O}(u_s)\right]. \tag{22}$$

Furthermore, since $\boldsymbol{p} \cdot \hat{\boldsymbol{x}} = 0$, we can write $\boldsymbol{p} = \phi_1(\boldsymbol{x})\hat{\boldsymbol{y}} + \phi_2(\boldsymbol{x})\hat{\boldsymbol{z}}$, for two scalar functions $\phi_1 = \mathcal{O}(u_s)$ and $\phi_2 = \mathcal{O}(u_s)$, which satisfy $\nabla^2\phi_1 = \nabla^2\phi_2 = 0$ subject to $\phi_1(\boldsymbol{x}), \phi_2(\boldsymbol{x}) \to 0$ as $|\boldsymbol{x}| \to \infty$ and

$$K\partial_r\phi_1 = W\left[(\phi_1 - u_s)\cos\eta + \phi_2\sin\eta\right]\cos\eta, \tag{23a}$$
$$K\partial_r\phi_2 = W\left[(\phi_1 - u_s)\cos\eta + \phi_2\sin\eta\right]\sin\eta, \tag{23b}$$

on $r = a$.

Free-free case: In the case of two free-filament ends, with $u_s(\pm L/2) = 0$, we can write u as a cosine series:

$$u(s,t) = \sum_{k=1}^{\infty} \hat{u}_k(t) \cos\left(\frac{q_k(2s+L)}{L}\right), \qquad [24]$$

where $q_k \equiv k\pi/2$. Using separation of variables, we pose general series representations for ϕ_1 and ϕ_2 of the form:

$$\phi_1(\boldsymbol{x}) = \sum_{k=1}^{\infty} \frac{-2q_k\hat{u}_k}{L} \sin\left(\frac{q_k(2s+L)}{L}\right)\left[\sum_{m=-\infty}^{\infty} \frac{K_m(2q_k r/L)}{K_m(2q_k a/L)} a_k^m e^{im\eta}\right], \qquad [25a]$$

$$\phi_2(\boldsymbol{x}) = \sum_{k=1}^{\infty} \frac{-2q_k\hat{u}_k}{L} \sin\left(\frac{q_k(2s+L)}{L}\right)\left[\sum_{m=-\infty}^{\infty} \frac{K_m(2q_k r/L)}{K_m(2q_k a/L)} b_k^m e^{im\eta}\right], \qquad [25b]$$

where $K_m(\cdot)$ is the mth modified Bessel function of the second kind and $a_k^m = \overline{a_k^{-m}}$ and $b_k^m = \overline{b_k^{-m}}$ are complex constants, which are to be determined now, and the bars denote complex conjugates.

The boundary conditions on $r = a$ impose the conditions

$$\sum_{m=-\infty}^{\infty} e^{im\eta}\left[\frac{S_k^m}{w} a_k^m + (a_k^m \cos\eta + b_k^m \sin\eta)\cos\eta\right] = \cos^2\eta, \qquad [26a]$$

$$\sum_{m=-\infty}^{\infty} e^{im\eta}\left[\frac{S_k^m}{w} b_k^m + (a_k^m \cos\eta + b_k^m \sin\eta)\sin\eta\right] = \cos\eta\sin\eta, \qquad [26b]$$

where $w = aW/K$ is the dimensionless anchoring strength, $S_k^m = S_m(2q_k a/L)$, and

$$S_m(x) \equiv -\frac{xK_m'(x)}{K_m(x)}. \qquad [27]$$

Replacing the trigonometric functions with exponentials and evaluating coefficients yields the conditions

$$2a_k^m\left[1 + \frac{2}{w}S_k^m\right] + a_k^{m-2} + a_k^{m+2} - ib_k^{m-2} + ib_k^{m+2} = 2\delta_{0m} + \delta_{2m} + \delta_{-2m}, \qquad [28a]$$

$$2ib_k^m\left[1 + \frac{2}{w}S_k^m\right] + a_k^{m-2} - a_k^{m+2} - ib_k^{m-2} - ib_k^{m+2} = \delta_{2m} - \delta_{-2m}. \qquad [28b]$$

where δ_{ij} is the Kronecker delta function. Solving these recurrence relations yields $a_k^m = b_k^m = 0$ for $m \notin \{-2, 0, 2\}$, $b_k^0 = 0$, $ib_k^2 = ib_k^{-2} = a_k^{-2} = a_k^2$,

$$a_k^0 = \frac{S_k^2}{S_k^0 + S_k^2 + \frac{2}{w}S_k^0 S_k^2}, \quad \text{and} \quad a_k^2 = \frac{S_k^0/2}{S_k^0 + S_k^2 + \frac{2}{w}S_k^0 S_k^2}. \qquad [29]$$

Altogether, we have the solution

$$\phi_1(\boldsymbol{x}) = \sum_{k=1}^{\infty} \frac{-2q_k\hat{u}_k}{L} \sin\left(\frac{q_k(2s+L)}{L}\right)\left[A_k \frac{K_0(2q_k r/L)}{K_0(2q_k a/L)} + B_k \cos(2\eta)\frac{K_2(2q_k r/L)}{K_2(2q_k a/L)}\right], \qquad [30a]$$

$$\phi_2(\boldsymbol{x}) = \sum_{k=1}^{\infty} \frac{-2q_k\hat{u}_k}{L} \sin\left(\frac{q_k(2s+L)}{L}\right)\left[B_k \sin(2\eta)\frac{K_2(2q_k r/L)}{K_2(2q_k a/L)}\right], \qquad [30b]$$

where

$$A_k \equiv \frac{1/S_0(2q_k a/L)}{1/S_2(2q_k a/L) + 1/S_0(2q_k a/L) + 2/w} \quad \text{and} \quad B_k \equiv \frac{1/S_2(2q_k a/L)}{1/S_2(2q_k a/L) + 1/S_0(2q_k a/L) + 2/w}. \qquad [31]$$

One free-end: In the case of one fixed and one free-filament ends, with $u(-L/2) = 0$ and $u_s(L/2) = 0$, respectively, we can instead write u as the series

$$u(s,t) = \sum_{k=1}^{\infty} \hat{u}_k(t) \sin\left(\frac{Q_k(2s+L)}{L}\right), \qquad [32]$$

for $Q_k \equiv (2k-1)\pi/4$. In this case, we find

$$\phi_1(\boldsymbol{x}) = \sum_{k=1}^{\infty} \frac{2Q_k\hat{u}_k}{L} \cos\left(\frac{Q_k(2s+L)}{L}\right)\left[C_k \frac{K_0(2Q_k r/L)}{K_0(2Q_k a/L)} + D_k \cos(2\eta)\frac{K_2(2Q_k r/L)}{K_2(2Q_k a/L)}\right], \qquad [33a]$$

$$\phi_2(\boldsymbol{x}) = \sum_{k=1}^{\infty} \frac{2Q_k\hat{u}_k}{L} \cos\left(\frac{Q_k(2s+L)}{L}\right)\left[D_k \sin(2\eta)\frac{K_2(2Q_k r/L)}{K_2(2Q_k a/L)}\right], \qquad [33b]$$

where

$$C_k \equiv \frac{1/S_0(Q_k 2a/L)}{1/S_2(2Q_k a/L) + 1/S_0(2Q_k a/L) + 2/w} \quad \text{and} \quad D_k \equiv \frac{1/S_2(2Q_k a/L)}{1/S_2(2Q_k a/L) + 1/S_0(2Q_k a/L) + 2/w}. \qquad [34]$$

Surface traction. To compute the traction on the filament due to the LC, we will need the Ericksen stress tensor and the surface stress tensor, given by

$$\mathbf{T} = \frac{K}{2}\|\nabla \boldsymbol{n}\|^2\mathbf{I} - K\nabla\boldsymbol{n}\cdot\nabla\boldsymbol{n}^T, \tag{35a}$$

$$\mathbf{T}_{\rm surf} = \mathcal{F}_{\rm surf}\mathbf{P}(\hat{\boldsymbol{\nu}}) - \mathbf{P}(\hat{\boldsymbol{\nu}})\cdot\frac{\partial\mathcal{F}_{\rm surf}}{\partial\hat{\boldsymbol{\nu}}}\hat{\boldsymbol{\nu}}, \tag{35b}$$

respectively, see (6, 7).

The first part of the traction is associated with the Ericksen stress $\mathbf{T}$, and is given by $\boldsymbol{f}(s,\eta) = \hat{\boldsymbol{\nu}}\cdot\mathbf{T}$. But this traction enters at $\mathcal{O}(\boldsymbol{p}^2) = \mathcal{O}(u_s^2)$, and so we neglect it. The second part of the traction is associated with the surface stress $\mathbf{T}_{\rm surf}$, and is given by $\boldsymbol{f}_{\rm surf}(s,\eta) = \nabla_s\cdot\mathbf{T}_{\rm surf}$, where $\nabla_s \equiv \mathbf{P}(\hat{\boldsymbol{\nu}})\cdot\nabla$ with $\mathbf{P}(\hat{\boldsymbol{\nu}}) \equiv \mathbf{I} - \hat{\boldsymbol{\nu}}\hat{\boldsymbol{\nu}}$, a projection operator for the surface normal direction. Since $\hat{\boldsymbol{\nu}} = \hat{\boldsymbol{r}} + \mathcal{O}(u_s)$, we note that

$$\nabla_s = \mathbf{P}(\hat{\boldsymbol{\nu}})\cdot\nabla = \left(\hat{\boldsymbol{x}}\partial_s + \frac{1}{r}\hat{\boldsymbol{\eta}}\partial_\eta\right)[1+\mathcal{O}(u_s)]. \tag{36}$$

For the weak degenerate tangential anchoring conditions considered above, we have the surface stress

$$\mathbf{T}_{\rm surf} = \frac{W}{2}(\boldsymbol{n}\cdot\hat{\boldsymbol{\nu}})^2\mathbf{P}(\hat{\boldsymbol{\nu}}) - W(\boldsymbol{n}\cdot\hat{\boldsymbol{\nu}})\mathbf{P}(\hat{\boldsymbol{\nu}})\cdot\boldsymbol{n}\hat{\boldsymbol{\nu}}, \tag{37}$$

for $\hat{\boldsymbol{\nu}} = \hat{\boldsymbol{r}} - u_s\cos\eta\hat{\boldsymbol{x}} + \mathcal{O}(u_s^2)$ and $\boldsymbol{n} = \hat{\boldsymbol{x}} + \phi_1\hat{\boldsymbol{y}} + \phi_2\hat{\boldsymbol{z}} + \mathcal{O}(u_s^2)$. It follows that $\boldsymbol{n}\cdot\hat{\boldsymbol{\nu}} = (\phi_1 - u_s)\cos\eta + \phi_2\sin\eta + \mathcal{O}(u_s^2)$, and so,

$$\mathbf{T}_{\rm surf} = -W[(\phi_1-u_s)\cos\eta + \phi_2\sin\eta]\hat{\boldsymbol{x}}\hat{\boldsymbol{r}}\,[1+\mathcal{O}(u_s)] = -K\partial_r\,[\phi_1\hat{\boldsymbol{x}}\hat{\boldsymbol{y}} + \phi_2\hat{\boldsymbol{x}}\hat{\boldsymbol{z}}]\,[1+\mathcal{O}(u_s)], \tag{38}$$

where we have used the boundary condition Eq. (22). Using Eq. (36) yields the surface traction:

$$\boldsymbol{f}_{\rm surf}(s,\eta) = \nabla_s\cdot\mathbf{T}_{\rm surf} = -K\,[\partial_s\partial_r\phi_1\hat{\boldsymbol{y}} + \partial_s\partial_r\phi_2\hat{\boldsymbol{z}}]\,[1+\mathcal{O}(u_s)]. \tag{39}$$

Finally, the force (per length) acting on the filament at each station s, i.e. $\boldsymbol{f}^{\rm LC}(s)$, is found by integrating the total surface traction, $\boldsymbol{f}(s,\eta) + \boldsymbol{f}_{\rm surf}(s,\eta)$, over the azimuthal angle, η:

$$\boldsymbol{f}^{\rm LC}(s) = \int_0^{2\pi}[\boldsymbol{f}(s,\eta)+\boldsymbol{f}_{\rm surf}(s,\eta)]\,a\mathrm{d}\eta = -aK\left[\left(\int_0^{2\pi}\partial_s\partial_r\phi_1\mathrm{d}\eta\right)\hat{\boldsymbol{y}} + \left(\int_0^{2\pi}\partial_s\partial_r\phi_2\mathrm{d}\eta\right)\hat{\boldsymbol{z}}\right][1+\mathcal{O}(u_s)]. \tag{40}$$

Note that $\hat{\boldsymbol{s}}\cdot\boldsymbol{f}^{\rm LC}(s) = \mathcal{O}(u_s^2)$, as used earlier.

Two free ends: For two free ends, we have from Eq. (30),

$$\partial_s\partial_r\phi_1 = \sum_{k=1}^\infty\left(\frac{2q_k}{L}\right)^2\frac{\hat{u}_k}{a}\cos\left(\frac{q_k(2s+L)}{L}\right)\left[A_kS_0\left(\frac{2q_ka}{L}\right) + B_k\cos(2\eta)S_2\left(\frac{2q_ka}{L}\right)\right], \tag{41a}$$

$$\partial_s\partial_r\phi_2 = \sum_{k=1}^\infty\left(\frac{2q_k}{L}\right)^2\frac{\hat{u}_k}{a}\cos\left(\frac{q_k(2s+L)}{L}\right)\left[B_k\sin(2\eta)S_2\left(\frac{2q_ka}{L}\right)\right], \tag{41b}$$

on $r=a$, for $q_k = \pi k/2$. Using that $\cos(2\eta)$ and $\sin(2\eta)$ have zero mean, and inserting the expressions in Eq. (31), the LC restoring force is found to be

$$\boldsymbol{f}^{\rm LC}(s) \sim -\frac{16\pi aK}{L^3}\hat{\boldsymbol{y}}\sum_{k=1}^\infty q_k^3\hat{u}_k\left[\frac{4q_ka}{wL} - \frac{K_0(2q_ka/L)}{K_0'(2q_ka/L)} - \frac{K_2(2q_ka/L)}{K_2'(2q_ka/L)}\right]^{-1}\cos\left(\frac{q_k(2s+L)}{L}\right). \tag{42}$$

Fixed-free ends: For only one free-end, the restoring force is similarly found to be Eq. (42), but with the substitutions $\cos\mapsto\sin$ and $q_k\mapsto Q_k = (2k-1)\pi/4$.

Filament dynamics and buckling length estimates. The filament dynamics in Eq. 18 are now fully determined, and we can begin to analyze them. Let us at this stage non-dimensionalize the problem by defining a dimensionless time, $\tau\equiv\alpha t$, dimensionless length, $\xi\equiv 2s/L(t)$, where $\xi\in[-1,1]$, and dimensionless displacement $U(\xi,\tau)\equiv u(s,t)/a$. We also introduce the dimensionless filament length $\Lambda(\tau)\equiv L(t)/(2a)$, dimensionless viscosity $\chi\equiv\zeta_\perp/\zeta_\parallel$, dimensionless anchoring strength $w\equiv aW/K$, and Ericksen number $\mathrm{Er}\equiv a^2\zeta_\parallel\alpha/(2\pi K)$.

From the uniform growth law, $L'(t) = \alpha L(t)$ (see the subsection on slender filament growth and kinematics above), we have that

$$\partial_t u(s,t) = a\alpha\,[\partial_\tau U(\xi,\tau) - \xi\partial_\xi U(\xi,\tau)], \tag{43}$$

and so the filament dynamics, i.e., Eq. 18, is governed by the dimensionless equation

$$\chi(U_\tau - \xi U_\xi) = \tilde{T}(\xi)U_{\xi\xi} + \mathrm{Er}^{-1}F^{\rm LC}(\xi,\tau), \tag{44}$$

for the dimensionless tension

$$\tilde{T}(\xi) \equiv \frac{T(s,t)}{\alpha\zeta_\parallel L(t)^2/4} = \begin{cases} -\left(1-\xi^2\right)/2 & \text{free-free,} \\ -\left(1-\xi\right)\left(3+\xi\right)/2 & \text{fixed-free,} \end{cases} \tag{45}$$

where we have dropped the end-force T_L as lower order, and inserted the dimensionless LC restoring force

$$F^{\rm LC}(\xi,\tau) \equiv \frac{f^{\rm LC}(s,t)}{2\pi K/a} = \begin{cases} -\displaystyle\sum_{k=1}^{\infty} \frac{q_k^3 U_k}{\Lambda^3}\left[\frac{2q_k}{w\Lambda} - \frac{K_0(q_k/\Lambda)}{K_0'(q_k/\Lambda)} - \frac{K_2(q_k/\Lambda)}{K_2'(q_k/\Lambda)}\right]^{-1} \cos\left[q_k(1+\xi)\right] & \text{free-free,} \\ -\displaystyle\sum_{k=1}^{\infty} \frac{Q_k^3 U_k}{\Lambda^3}\left[\frac{2Q_k}{w\Lambda} - \frac{K_0(Q_k/\Lambda)}{K_0'(Q_k/\Lambda)} - \frac{K_2(Q_k/\Lambda)}{K_2'(Q_k/\Lambda)}\right]^{-1} \sin\left[Q_k(1+\xi)\right] & \text{fixed-free,} \end{cases} \tag{46}$$

with $q_k = k\pi/2$, $Q_k = (2k-1)\pi/4$, and the Fourier series

$$U(\xi,\tau) = \begin{cases} \displaystyle\sum_{k=1}^{\infty} U_k(\tau)\cos\left[q_k(1+\xi)\right] & \text{free-free,} \\ \displaystyle\sum_{k=1}^{\infty} U_k(\tau)\sin\left[Q_k(1+\xi)\right] & \text{fixed-free.} \end{cases} \tag{47}$$

with coefficients $U_k(\tau) \equiv u_k(t)/a$.

Two free-ends. In the free-free case, inserting the Fourier series for $U(\xi,\tau)$ and using the orthogonality of the basis functions yields

$$\chi U_k'(\tau) = \left\{\frac{\chi}{2} + \frac{q_k^2}{3} - \frac{1}{4} - \frac{q_k^3}{{\rm Er}\Lambda^3} Z_k^{-1}\right\} U_k(\tau) + \sum_{m\neq k} J_{km} U_m(\tau), \tag{48}$$

for $q_k = \pi k/2$, where

$$Z_k \equiv \frac{2q_k}{w\Lambda} - \frac{K_0(q_k/\Lambda)}{K_0'(q_k/\Lambda)} - \frac{K_2(q_k/\Lambda)}{K_2'(q_k/\Lambda)}, \tag{49}$$

is the mode-k coefficient appearing in main text Eq. 4, with the fixed-free counterpart obtained by $q_k \mapsto Q_k$; and

$$\begin{aligned} J_{km} &= \int_{-1}^{1} \left\{\frac{q_m^2}{2}(1-\xi^2)\cos\left[q_m(1+\xi)\right] - \chi q_m \xi \sin\left[q_m(1+\xi)\right]\right\} \cos\left[q_k(1+\xi)\right] {\rm d}\xi \\ &= -\left[1+(-1)^{k+m}\right] \frac{m^2\left[m^2(1-\chi)+k^2(1+\chi)\right]}{(k^2-m^2)^2}, \end{aligned} \tag{50}$$

when $m \neq k$. Here, we see that the even and odd modes about $\xi = 0$ have uncoupled. Since there is fairly rapid decay in k away from $k = m$, a first approximation is to ignore the summation in Eq. 48. Doing this yields

$$\chi U_k'(\tau) \approx \left\{\frac{\chi}{2} + \frac{q_k^2}{3} - \frac{1}{4} - \frac{q_k^3}{{\rm Er}\Lambda^3} Z_k^{-1}\right\} U_k(\tau). \tag{51}$$

Although $\Lambda(\tau)$ is generally time-dependent, to find the buckling lengths, we shall freeze it at its instantaneous value, $\Lambda(\tau) = \Lambda$, and pose the ansatz $U_k(\tau) = e^{\sigma_k \tau}\hat{U}_k$, yielding the growth rate of the kth mode:

$$\sigma_k \approx \frac{1}{2} + \frac{1}{\chi}\left\{\frac{q_k^2}{3} - \frac{1}{4} - \frac{q_k^3}{{\rm Er}\Lambda^3} Z_k^{-1}\right\}. \tag{52}$$

It follows that the kth mode becomes unstable when $\Lambda > \Lambda_k$ where Λ_k solves

$$\frac{\chi}{2} + \frac{q_k^2}{3} - \frac{1}{4} - \frac{q_k^3}{{\rm Er}\Lambda_k^3}\left[\frac{2q_k}{w\Lambda_k} - \frac{K_0(q_k/\Lambda_k)}{K_0'(q_k/\Lambda_k)} - \frac{K_2(q_k/\Lambda_k)}{K_2'(q_k/\Lambda_k)}\right]^{-1} \approx 0. \tag{53}$$

For small Er (and thus large Λ_k), we note that

$$\frac{\Lambda_k^3}{q_k^3}\left[\frac{2q_k}{w\Lambda_k} - \frac{K_0(q_k/\Lambda_k)}{K_0'(q_k/\Lambda)} - \frac{K_2(q_k/\Lambda_k)}{K_2'(q_k/\Lambda_k)}\right] = \frac{\Lambda_k^2}{2q_k^2}\left(1 - 2\gamma + \frac{4}{w} + \log\frac{4\Lambda_k^2}{q_k^2}\right) + \mathcal{O}(1), \tag{54}$$

as $q_k/\Lambda_k \to 0$, where γ is the Euler–Mascheroni constant. At leading-order in ${\rm Er}^{-1}$, assumed large, we have that

$$\frac{\Lambda_k^2}{2q_k^2}\left(1 - 2\gamma + \frac{4}{w} + \log\frac{4\Lambda_k^2}{q_k^2}\right) \approx \frac{4{\rm Er}^{-1}}{2\chi - 1 + 4q_k^2/3}, \tag{55}$$

which finally gives the approximation

$$\Lambda_k \approx \left(\frac{2\pi^2k^2\mathrm{Er}^{-1}}{2\chi - 1 + \pi^2k^2/3}\right)^{1/2}\left[W\left(\frac{32\mathrm{Er}^{-1}e^{1-2\gamma+4/w}}{2\chi - 1 + \pi^2k^2/3}\right)\right]^{-1/2}, \qquad [56]$$

where $W(z)$ is the Lambert W function (the solution to $W \exp W = z$). Using the rudimentary approximation of $W(z) \approx \log z$ for large z, this expression can be approximated further as

$$\Lambda_k \approx \left(\frac{2\pi^2k^2\mathrm{Er}^{-1}}{2\chi - 1 + \pi^2k^2/3}\right)^{1/2}\left[\log\left(\frac{32\mathrm{Er}^{-1}}{2\chi - 1 + \pi^2k^2/3}\right) + 1 - 2\gamma + \frac{4}{w}\right]^{-1/2}. \qquad [57]$$

The buckling length of the filament is then given by the smallest critical length, $\Lambda^* = \min_k \Lambda_k$. For $\chi = 5$, $\mathrm{Er} = 10^{-6}$, and $w = 10^{-2}$–10^0, we find that the smallest mode is $k = 1$, which gives the estimate to dimensionless buckling $\Lambda^* \approx 62.2$–294. This approximation is compared to numerical solutions of the coupled system, Eq. 48, in the main text Fig. 5. Although the numerical results are not quantitatively recovered, the approximation does recover the qualitative behavior. It thus inspires the fitting expression presented in the main text:

$$\Lambda^* = \left(\frac{a_1\mathrm{Er}^{-1}}{\log(a_2\mathrm{Er}^{-1}) + 4/w}\right)^{1/2}, \qquad [58]$$

for fitting parameters a_i.

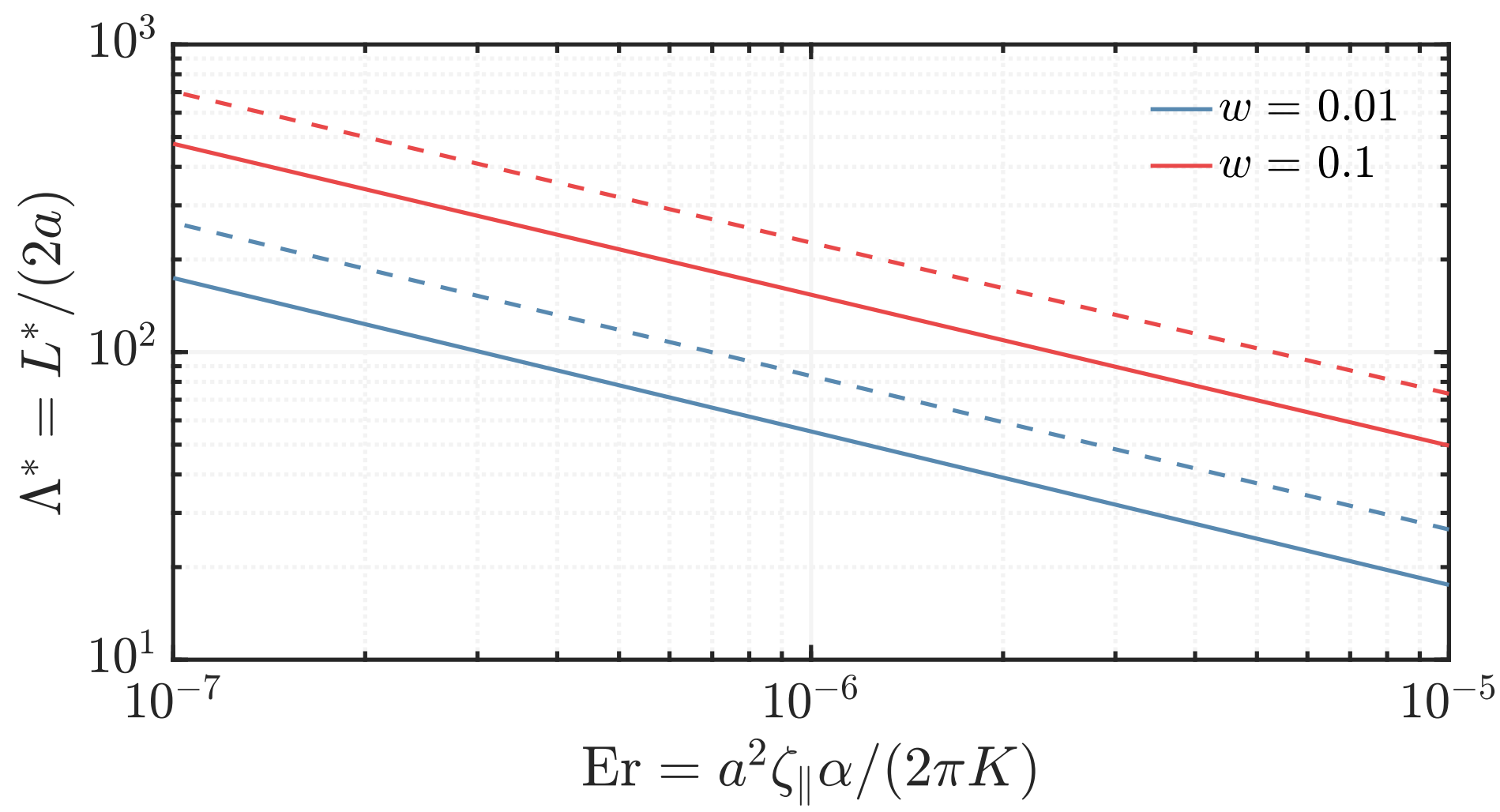

Fig. S1. The analytical approximation [Eq. 58] for the dimensionless buckling length, Λ^*, as a function of the Ericksen number, Er, for free-free (dashed curves) and fixed-free (solid curves) boundary conditions. A fixed end reduces the buckling length by a factor of ≈ 1.5.

The most unstable waveforms predicted by the theory are found by inserting the ansatz $U(\xi,\tau) = U_i(\xi)\exp(\sigma_i\tau)$ into Eq. (44), and solving for the eigenpairs $(U_i(\xi), \sigma_i)$. The eigenfunctions are normalized such that $\|U_i\|_\infty = 1$. The most unstable waveform is shown in Fig. S2(a) for a range of dimensionless filament lengths. These waveforms generically involve all of the available Fourier modes due to their coupling through the spatially varying tension.

As discussed above, for lengths just beyond the critical buckling length, the most unstable waveforms are approximately the lowest Fourier mode that respects the boundary conditions ($k = 1$). For longer filaments, the most unstable mode becomes oscillatory, and localized near the filament center, where the compressive load is strongest. This phenomenon is comparable to the buckling of an elastic beam with spatially varying thickness (9), and of other inhomogeneous structures (10).

Similar to the theoretical predictions, the experiments show spatially localized, oscillatory modes after the onset of buckling. But the experiments do not show the increasingly high wavenumber modes predicted by the theory at yet larger filament lengths. A fully nonlinear theory, that allows for large displacements, is needed to explore this post-buckling regime. The theory is made considerably more challenging in this case as the LC director field is then governed by a nonlinear PDE, which requires an extended study.

The growth rates corresponding to the most unstable eigenmodes, denoted $\sigma_{\max}$, are shown in Fig. S2(b). Note that these are real and positive for sufficiently long filaments, indicating its susceptibility to buckle. Furthermore, the growth rates are notably non-monotonic in the filament length after the buckling threshold has been crossed, but before the localized, oscillatory modes emerge.

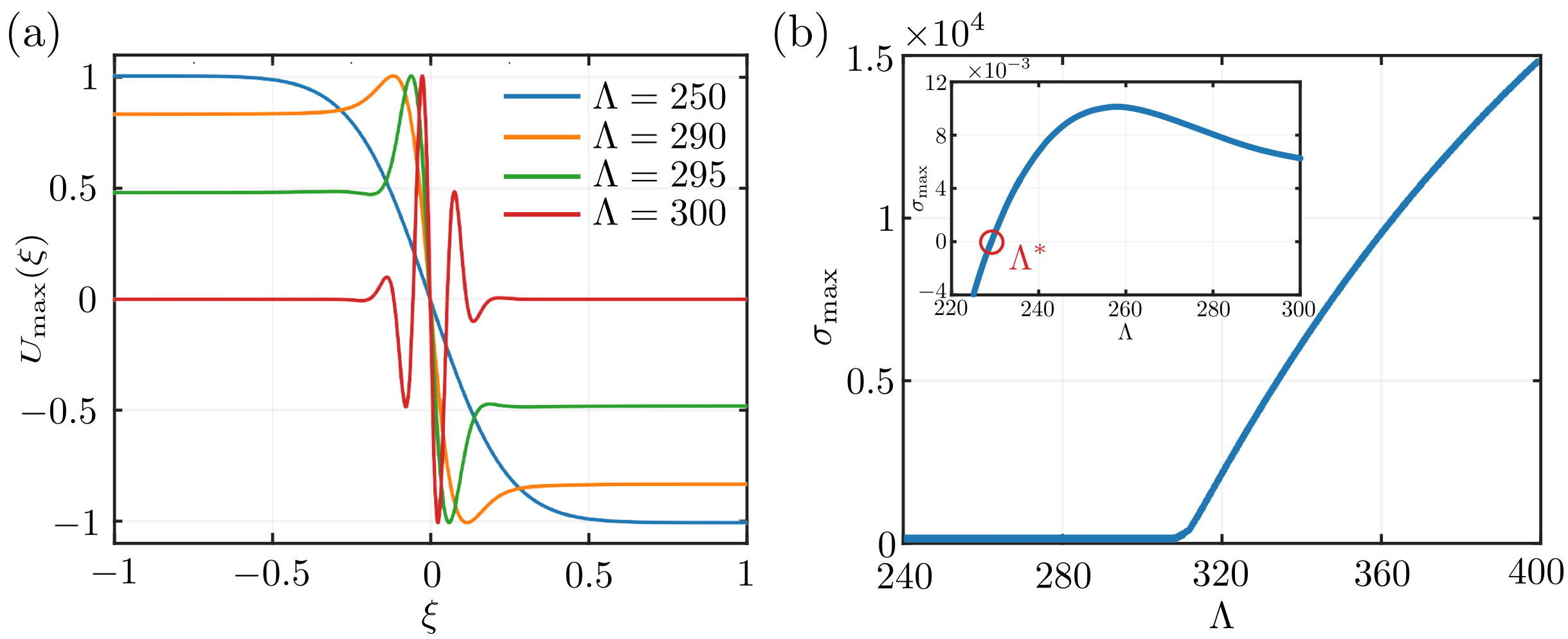

Fig. S2. (a) The most unstable eigenfunction, $U_{\max}(\xi)$, for $\chi = 5$, $\mathrm{Er} = 10^{-6}$, $w = 0.1$, and free-free boundary conditions. The first unstable mode has a single long wavelength. For longer filaments, the most unstable mode is highly oscillatory and spatially-localized near the filament center, akin to the shapes observed in the experiments. (b) The corresponding growth rates, $\sigma_{\max}$, are non-monotonic in the filament length for values above the critical buckling length, Λ^*, but below that for which localized oscillatory modes appear.

Fixed-free ends. In the fixed-free case, inserting the Fourier series for $U(\xi,\tau)$ and using the orthogonality of the basis functions yields a similar expression to before:

$$\chi U_k'(\tau) = \left\{ \frac{4Q_k^2}{3} - \frac{1}{4} - \frac{Q_k^3}{\mathrm{Er}\Lambda^3}\left[\frac{2Q_k}{w\Lambda} - \frac{K_0(Q_k/\Lambda)}{K_0'(Q_k/\Lambda)} - \frac{K_2(Q_k/\Lambda)}{K_2'(Q_k/\Lambda)}\right]^{-1}\right\} U_k(\tau) + \sum_{m\neq k} I_{km} U_m(\tau), \tag{59}$$

for $Q_k = \pi(2k-1)/4$ and

$$\begin{aligned} I_{km} &= \int_{-1}^{1} \left\{ \frac{Q_m^2}{2}(1-\xi)(3+\xi)\sin\left[Q_m(1+\xi)\right] + \chi Q_m \xi \cos\left[Q_m(1+\xi)\right]\right\} \sin\left[Q_k(1+\xi)\right] \mathrm{d}\xi \\ &= -\frac{(-1)^{k+m}(2m-1)^2\left[(2k-1)^2+(2m-1)^2\right]}{8(k-m)^2(k+m-1)^2} - \frac{(2m-1)\left[2k-1+(-1)^{k+m}(2m-1)\right]\chi}{4(k-m)(k+m-1)}, \end{aligned} \tag{60}$$

when $m \neq k$.

Again, omitting the summation in Eq. 59 and taking the ansatz $U_k(\tau) = e^{\sigma_k \tau}\hat{U}_k$ yields an approximation for the growth rate of the kth mode,

$$\sigma_k \approx \frac{1}{\chi}\left\{ \frac{4Q_k^2}{3} - \frac{1}{4} - \frac{Q_k^3}{\mathrm{Er}\Lambda^3}\left[\frac{2Q_k}{w\Lambda} - \frac{K_0(Q_k/\Lambda)}{K_0'(Q_k/\Lambda)} - \frac{K_2(Q_k/\Lambda)}{K_2'(Q_k/\Lambda)}\right]^{-1}\right\}. \tag{61}$$

For small Er, making the same approximations as before yields the fact that the kth mode becomes unstable when $\Lambda > \Lambda_k$, where

$$\Lambda_k \approx \left(\frac{(2k-1)^2\pi^2\mathrm{Er}^{-1}/2}{\pi^2(2k-1)^2/3-1}\right)^{1/2}\left[W\left(\frac{32\mathrm{Er}^{-1}}{\pi^2(2k-1)^2/3-1}e^{1-2\gamma+4/w}\right)\right]^{-1/2}, \tag{62}$$

or, with the additional rudimentary approximation,

$$\Lambda_k \approx \left(\frac{(2k-1)^2\pi^2\mathrm{Er}^{-1}/2}{\pi^2(2k-1)^2/3-1}\right)^{1/2}\left[\log\left(\frac{32\mathrm{Er}^{-1}}{\pi^2(2k-1)^2/3-1}\right)+1-2\gamma+\frac{4}{w}\right]^{-1/2}. \tag{63}$$

For $\chi = 5$, $\mathrm{Er} = 10^{-6}$, and $w = 10^{-2}$–10^{0}, here the smallest value of Λ_k is found to range between when $k = 6$ (for $w = 10^{-2}$) and $k = 2$ (for $w = 10^{0}$), giving the estimate to dimensionless buckling $\Lambda^* \approx 60.5$–296. An improved estimation is found by fitting Eq. (58) to the coupled system, as presented in the main text.

For lengths beyond the critical buckling length, Λ^*, the most unstable eigenmodes are again oscillatory, as in Fig. S2(a), but are localized near the pinned end. Unlike in the free-free case, however, there does not appear to be an unstable small wavenumber mode that emerges before the localized, oscillatory modes are triggered.

Supplementary Movies

Movie S1. Non-motile *E. coli* proliferating in LB media without DSCG.

Movie S2. Non-motile *E. coli* proliferating in LB media supplemented with $15\,\mathrm{w/w\%}$ DSCG.

Movie S3. Non-motile *E. coli* proliferating in LB media supplemented with $15\,\mathrm{w/w\%}$ DSCG. At $t = 0$ min we flush a suspension of chains with LC free liquid at a flow rate of $2.5\,\mathrm{\mu L/min}$, thus decreasing the LC concentration as time increases.

Movie S4. Non-motile *E. coli* proliferating in LB media supplemented with $15\,\mathrm{w/w\%}$ DSCG. At $t = 0$ min we flush a suspension of chains with $15\,\mathrm{w/w\%}$ DSCG solution at a flow rate of $2.5\,\mathrm{\mu L/min}$ keeping the LC concentration constant as time increases.

References

1. P van der Schoot, Depletion interactions in lyotropic nematics. *The J. Chem. Phys.* **112**, 9132–9138 (2000).
2. HNW Lekkerkerker, *Colloids and the depletion interaction / Henk N. W. Lekkerkerker, Remco Tuinier.* (Springer, Dordrecht ;), (2011) Series Title: Lecture notes in physics, v. 833 Publication Title: Colloids and the depletion interaction.
3. T Vissers, et al., Bacteria as living patchy colloids: Phenotypic heterogeneity in surface adhesion. *Sci. Adv.* **4**, eaao1170 (2018).
4. A Stepulane, AK Rajasekharan, M Andersson, Multifunctional surface modification of pdms for antibacterial contact killing and drug-delivery of polar, nonpolar, and amphiphilic drugs. *ACS Appl. Bio Mater.* **5**, 5289–5301 (2022).
5. BJ Walker, K Ishimoto, H Gadêlha, EA Gaffney, Filament mechanics in a half-space via regularised stokeslet segments. *J. Fluid Mech.* **879**, 808–833 (2019).
6. PG De Gennes, J Prost, *The physics of liquid crystals.* (Oxford university press) No. 83, (1993).
7. TGJ Chandler, SE Spagnolie, Rigid and deformable bodies in nematic liquid crystals. *Phys. Rev. Fluids* **9**, 110511 (2024).
8. A Rapini, M Papoular, Distorsion d'une lamelle nématique sous champ magnétique conditions d'ancrage aux parois. *J. Phys. Colloq.* **30**, C4–54 (1969).
9. A Luongo, Mode localization in dynamics and buckling of linear imperfect continuous structures. *Nonlinear Dyn.* **25**, 133–156 (2001).
10. CG Johnson, U Jain, AL Hazel, D Pihler-Puzović, T Mullin, On the buckling of an elastic holey column. *Proc. Royal Soc. A: mathematical, physical engineering sciences* **473** (2017).
11. A Habibi, C Blanc, NB Mbarek, T Soltani, Passive and active microrheology of a lyotropic chromonic nematic liquid crystal disodium cromoglycate. *J. Mol. Liq.* **288**, 111027 (2019).
12. I Duchesne, S Rainville, T Galstian, Bacterial motility reveals unknown molecular organization. *Biophys. journal* **109**, 2137–2147 (2015).
13. M Gómez-González, JC Del Alamo, Two-point particle tracking microrheology of nematic complex fluids. *Soft matter* **12**, 5758–5779 (2016).
14. PC Mushenheim, RR Trivedi, HH Tuson, DB Weibel, NL Abbott, Dynamic self-assembly of motile bacteria in liquid crystals. *Soft Matter* **10**, 88–95 (2014).
15. S Zhou, et al., Dynamic states of swimming bacteria in a nematic liquid crystal cell with homeotropic alignment. *New J. Phys.* **19**, 055006 (2017).
16. H Chi, M Potomkin, L Zhang, L Berlyand, IS Aranson, Surface anchoring controls orientation of a microswimmer in nematic liquid crystal. *Commun. Phys.* **3**, 162 (2020).
17. S Zhou, A Sokolov, OD Lavrentovich, IS Aranson, Living liquid crystals. *Biophys. J.* **106**, 420a (2014).
18. S Zhou, et al., Elasticity, viscosity, and orientational fluctuations of a lyotropic chromonic nematic liquid crystal disodium cromoglycate. *Soft Matter* **10**, 6571–6581 (2014).

Table S1. Summary of measurements and parameters used in theoretical calculations.

Parameter description	Symbol	Value
Cell growth rate	α	$2 \times 10^{-4}\,\mathrm{s}^{-1}$
DSCG parallel viscosity	$\mu_{\parallel}$	$0.4\,\mathrm{Pa}\cdot\mathrm{s}$ (11) $(18\,\mathrm{w/w\%})$<br>$0.1\,\mathrm{Pa}\cdot\mathrm{s}$ (11) $(15\,\mathrm{w/w\%})$
DSCG viscosity ratio	$\mu_{\perp}/\mu_{\parallel}$	2–6 (measured, cf. (12, 13))
Cell radius	a	$1\,\mu\mathrm{m}$
LC–bacteria surface anchoring energy	W	$10^{-5}\,\mathrm{J/m^2}$ (14)<br>$10^{-6}\,\mathrm{J/m^2}$ (15)<br>$0.2 \times 10^{-5}\,\mathrm{J/m^2}$ (16)
DSCG average Frank elastic constant	K	$18.66\,\mathrm{pN}$ (17) $(18\,\mathrm{w/w\%})$<br>$11.91\,\mathrm{pN}$ (17) $(15\,\mathrm{w/w\%})$
Volume fraction	ϕ	0.115 (18)
Average LC molecule diameter	D	$1.6\,\mathrm{nm}$ (18)
Average LC molecule length	l	$20\,\mathrm{nm}$ (18)
Cell length	l_{b}	1–$4\,\mu\mathrm{m}$
Buckling length	L^{*}	$(87 \pm 42)\,\mu\mathrm{m}$ $(18\,\mathrm{w/w\%}$ DSCG)<br>$(166 \pm 93)\,\mu\mathrm{m}(15\,\mathrm{w/w\%}$ DSCG)
cell height from surface	h	$(1.0 \pm 0.4)\,\mu\mathrm{m}$ $(18\,\mathrm{w/w\%}$ DSCG)<br>$(1.0 \pm 0.7)\,\mu\mathrm{m}$ $(15\,\mathrm{w/w\%}$ DSCG)

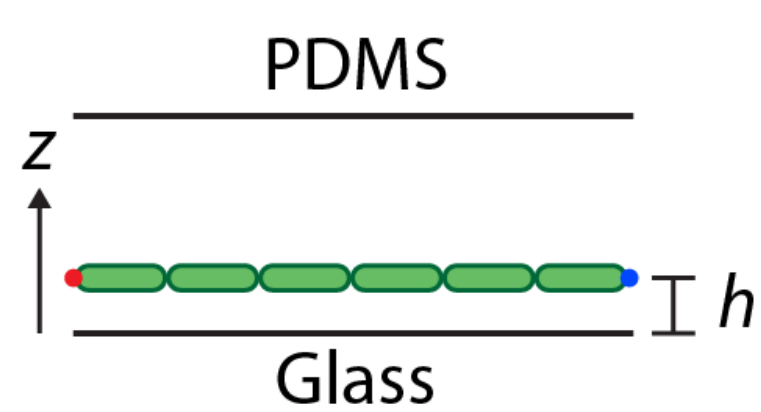

Fig. S3. Schematic depicting the geometry to estimate the distance between a bacterial chain and a surface. The schematic shows a bacterial chain (green spherocylinders) within the custom PDMS device. The bacterial chain is a distance h from the glass bottom surface. To estimate h we track the endpoints of the bacterial chain (indicated here as red and blue dots). Next, we take the standard deviation of the z-position time series to get an estimate of the chain's fluctuations near the surface of the imaging device. The average of the fluctuations yields an estimate of h for a single chain. We repeat this process for multiple buckling chains to obtain the average values shown in Table S1.

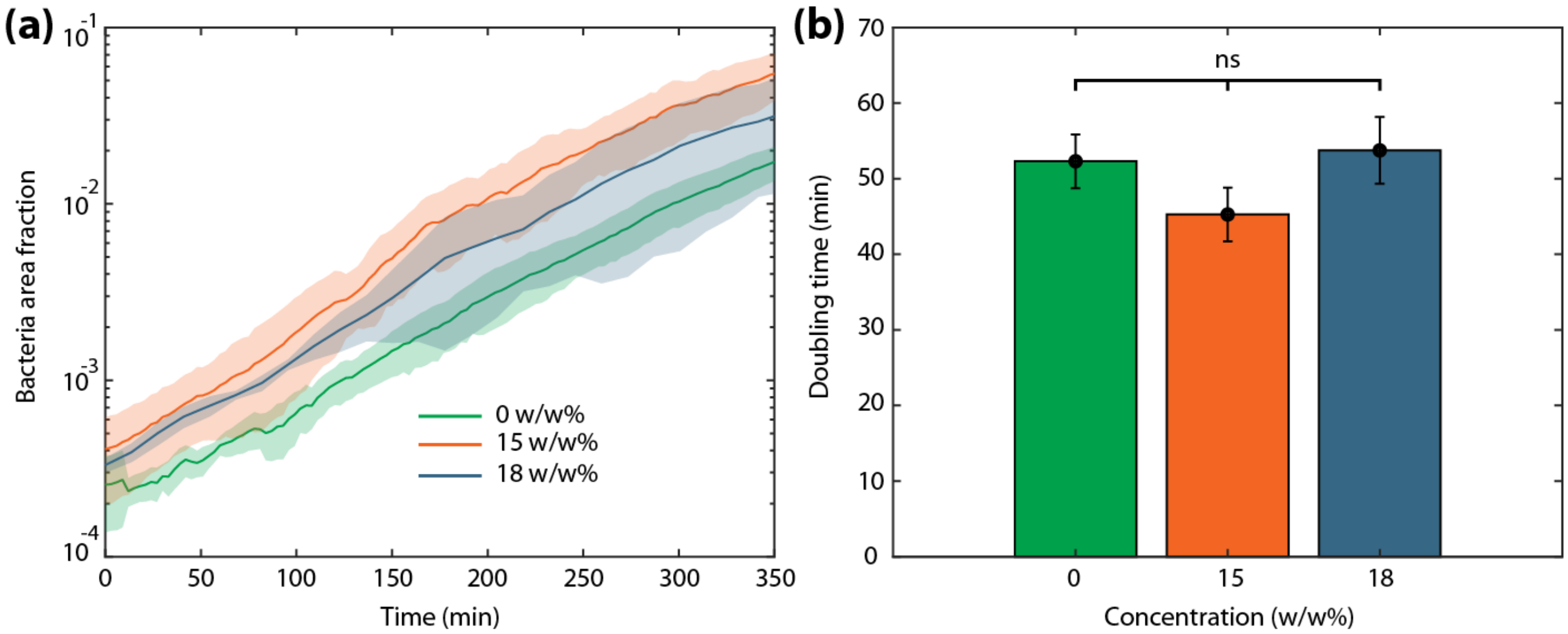

Fig. S4. ***E. coli*** **doubling times are similar across different LC conditions**. **(A)** and **(B)** show the growth curves and doubling times for *E. coli* cells in different LC solutions. The growth curves were obtained in triplicate where the solid curve represents the mean among all samples while the shaded regions are the corresponding standard deviations. The doubling times were measured by fitting the first 500 min of each sample's growth curve to an exponential equation: $A_{\mathrm{frac}} = A_0 e^{\alpha t}$ where t is time, A_0 is the area fraction at $t = 0$, α is the maximal growth rate, and A_{frac} is the cell collective's total area fraction. We convert the bacteria's maximal growth rate, α, to a doubling time via $t_{\mathrm{d}} = \log(2)/\alpha$. We observe that, throughout all LC concentrations tested, *E. coli*'s doubling time remains between 52–68 min and does not depend appreciably on LC concentration. We obtained the growth curves under different LC concentrations via segmentation of images in MATLAB to then plot the area fraction that cells occupy as a function of time.

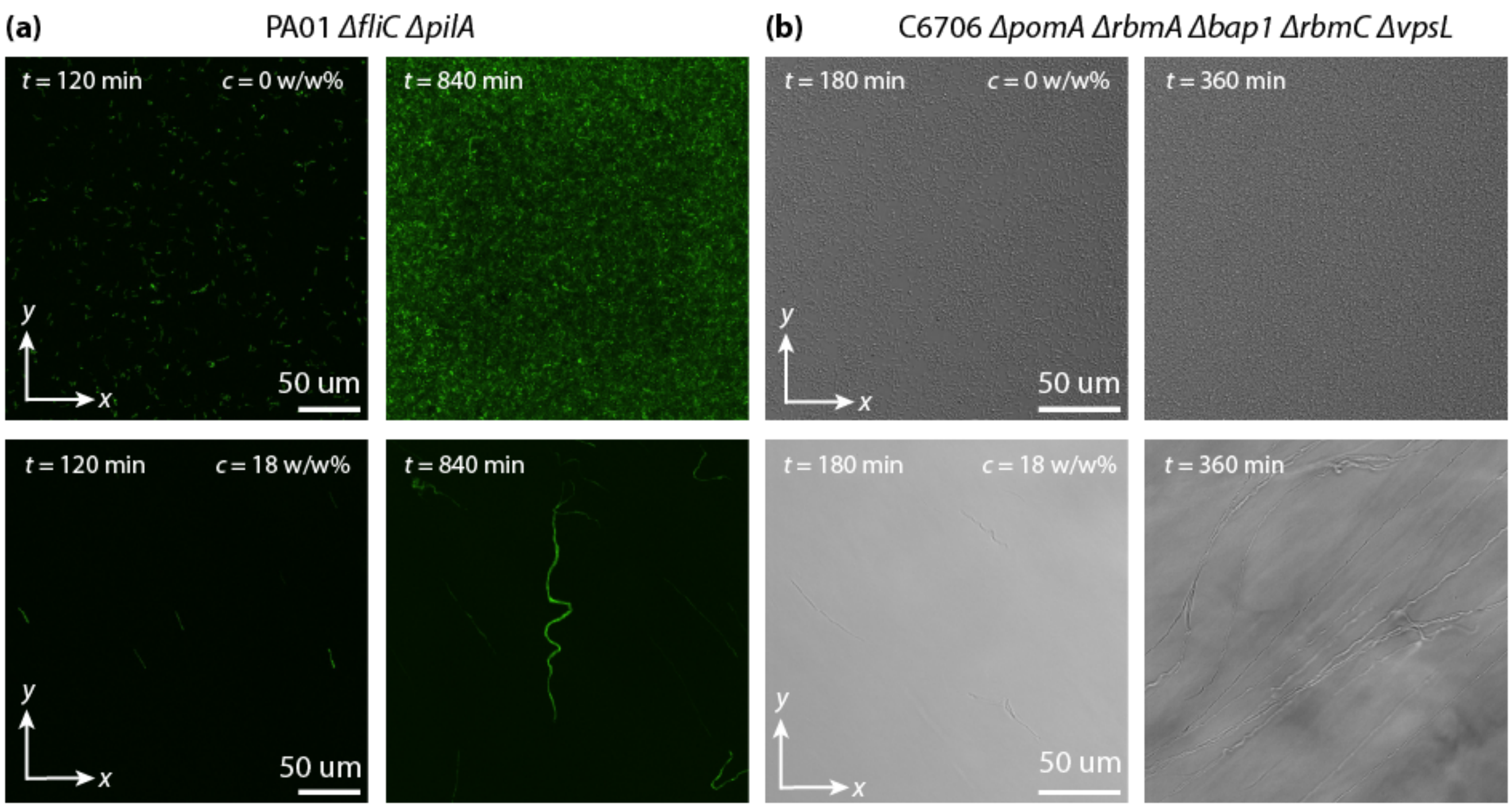

Fig. S5. Nonmotile *P. aeruginosa* and *V. cholerae* form chains as they proliferate in liquid crystalline solutions. (A) Time sequence of nonmotile *P. aeruginosa* proliferating in nutrient-rich fluid without (top) or with (bottom) $18\,\mathrm{w/w\%}$ DSCG. **(B)** Same as in **A** but with nonmotile *V. cholerae*. The time indicates the duration elapsed after the 96-well plate containing the samples is inserted into the static incubator. The images were taken at different positions of the same well at the different time points.

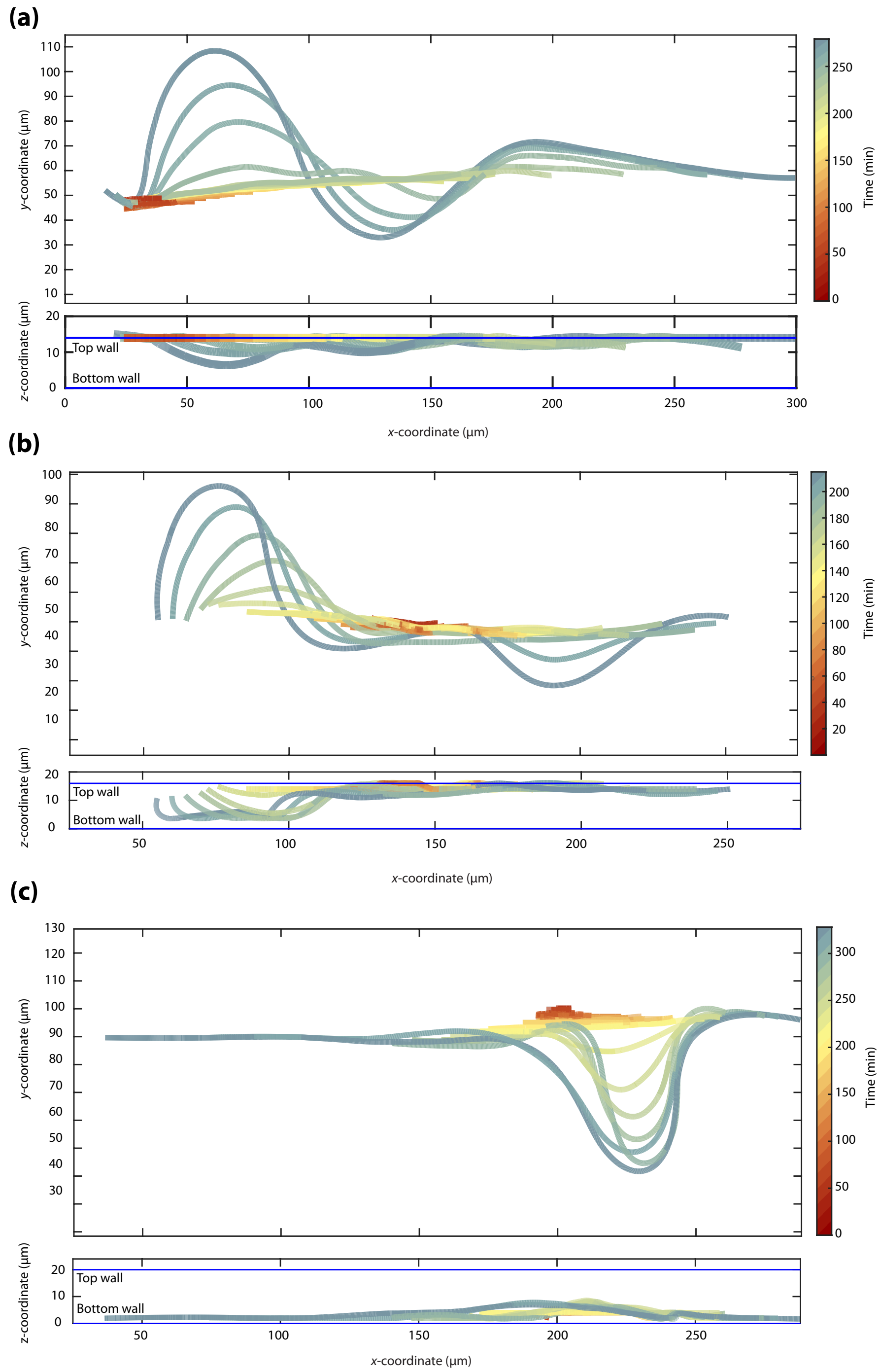

Fig. S6. Growing bacterial chains buckle near the top and bottom walls of the imaging device. (a)–(c) Three-dimensional reconstructions of representative single growing bacterial chains buckling in $18\,\mathrm{w/w\%}$ DSCG; colors indicate time. In each panel, the upper plot shows the chain centerline projected onto the xy-plane and the lower plot shows the same centerline projected onto the xz-plane. The blue horizontal lines mark the top and bottom walls of the imaging device. In all cases, the chain elongates parallel to and within $\approx 1\,\mu\mathrm{m}$ of a wall, and the buckling deformation develops predominantly in the plane parallel to the wall.

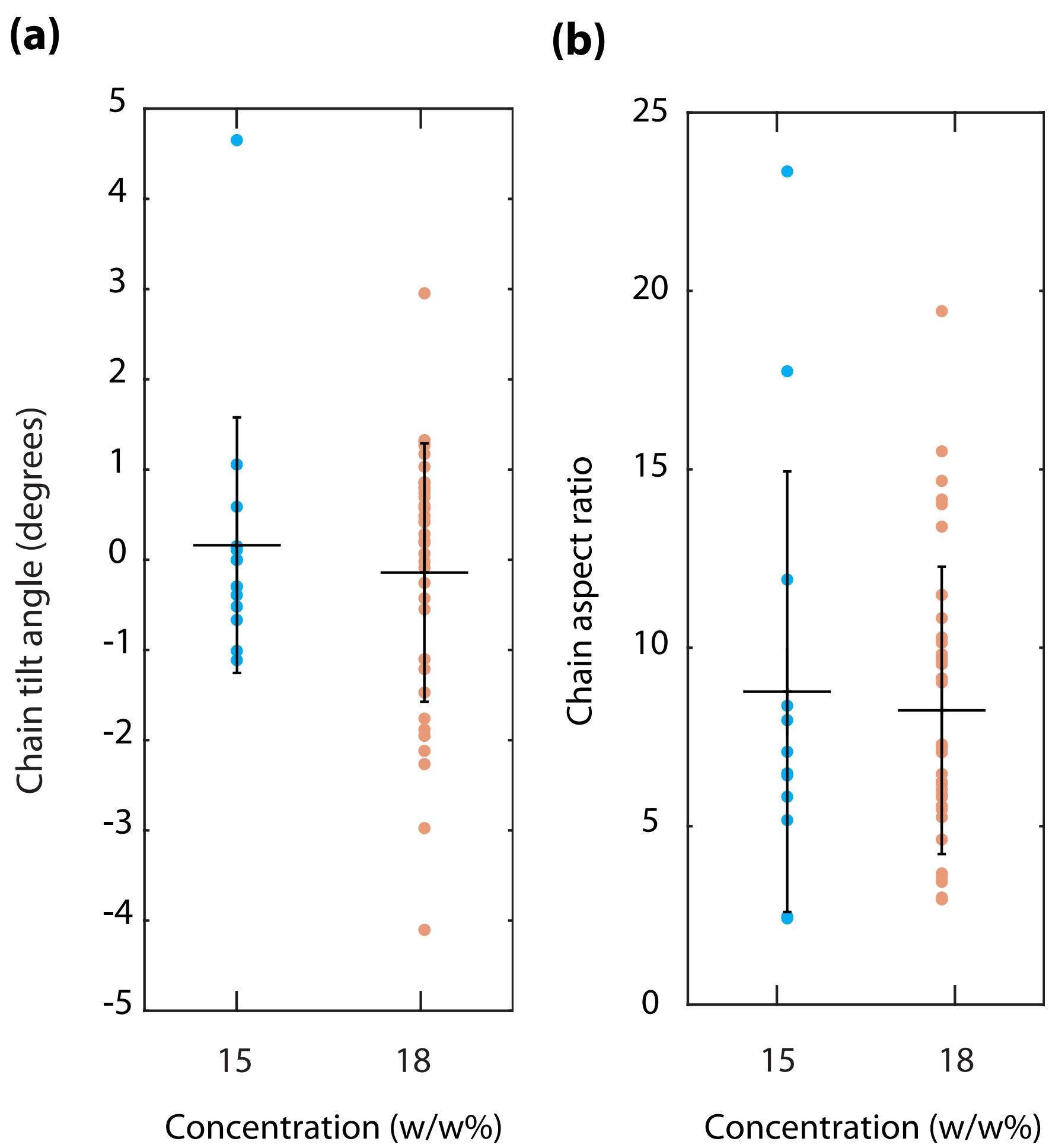

Fig. S7. Growing bacterial chains elongate horizontally and buckle within the xy-plane. (a) Tilt angle of buckled chains in $15\,\mathrm{w/w\%}$ (light blue, $n = 13$) and $18\,\mathrm{w/w\%}$ (light orange, $n = 38$) DSCG. The tilt angle is the angle between the principal axis of an ellipsoid encapsulating the chain (oriented along the chain's end-to-end vector) and the xy-plane. Horizontal lines indicate means close to zero ($0.1°$ and $-0.2°$, respectively), indicating that chains grow horizontally, parallel to the walls. **(b)** Aspect ratio of the same chains at long times, defined as the ratio of the semi-axes of the encapsulating ellipsoid lying in the xy- and xz-planes. Horizontal lines indicate means; aspect ratios well above unity (≈ 8–9) indicate that the buckled conformation is largely contained in the xy-plane. Error bars indicate standard deviations.

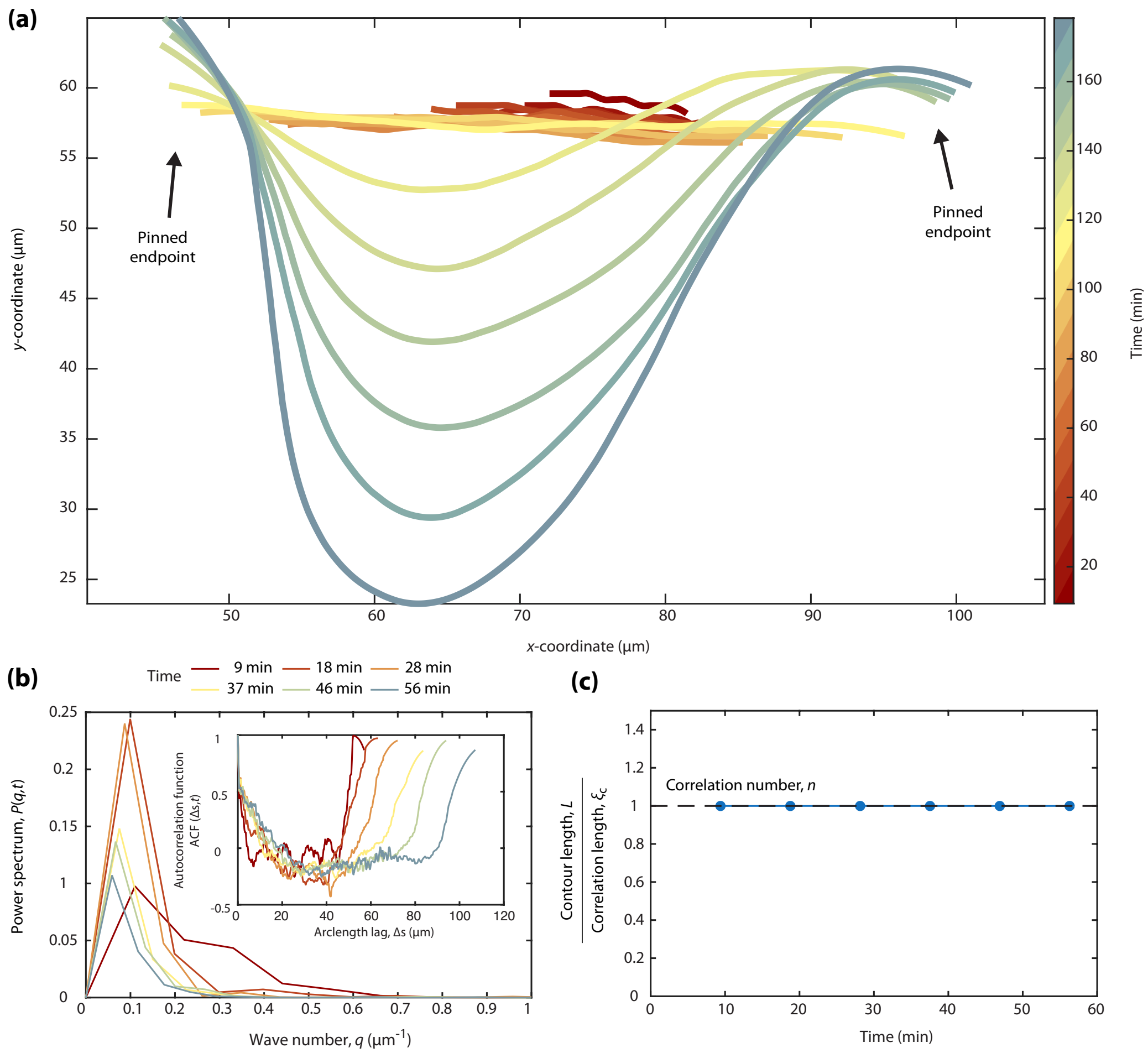

Fig. S8. A bacterial chain pinned at both endpoints exhibits a single long-wavelength deformation. (a) Successive centerline configurations of a buckling chain in $18\,\mathrm{w/w\%}$ DSCG; colors indicate time. The chain initially grows straight and then develops a single, long-wavelength arc rather than multiple localized buckles. Arrows mark the chain's two pinned endpoints. **(b)** Power spectrum $P(q,t)$ of the chain in **(a)** after the onset of buckling; the wavenumber at which P is maximal defines the dominant wavenumber q_c. The inset shows the corresponding curvature autocorrelation function $\mathrm{ACF}(\Delta s, t)$; its rise at the largest arclength lags is an expected feature of the unbiased estimator, for which progressively fewer segment pairs contribute as the lag approaches the contour length. The correlation length is $\xi = 2\pi/q_c$. **(c)** Time evolution of the ratio of the contour length L to the correlation length ξ; at long times the ratio saturates at the correlation number $n = 1$, close to the $n \to 1$ limit expected for a single arc. This long-wavelength deformation, distinct from the localized buckling of less-constrained chains, illustrates how strong pinning at both ends suppresses the localized, short-wavelength modes.

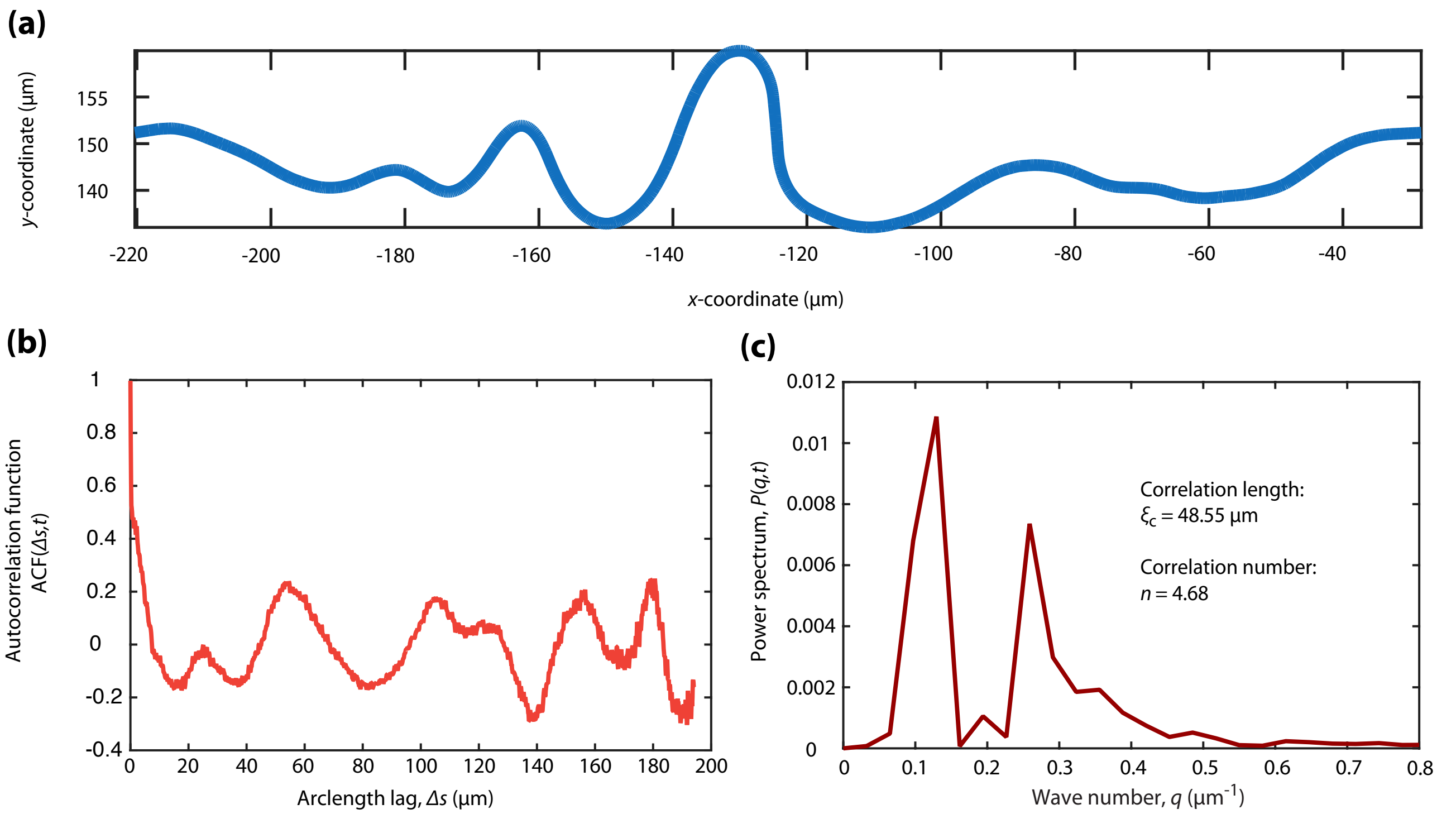

Fig. S9. Nonmotile *P. aeruginosa* chains exhibit localized buckling. **(a)** Centerline configuration of a buckled chain in $18\,\mathrm{w/w\%}$ DSCG. **(b)** Curvature autocorrelation function $\mathrm{ACF}(\Delta s, t)$ of the chain in **(a)**, computed via Eq. 2. **(c)** Power spectrum $P(q, t)$ of the chain in **(a)** after the onset of buckling, computed via Eq. 3; the wavenumber at which P is maximal defines the dominant wavenumber q_c. The correlation length is $\xi_\mathrm{c} = 2\pi/q_\mathrm{c} = 48.55\,\mu\mathrm{m}$, giving a correlation number $n = L/\xi_\mathrm{c} = 4.68 > 1$, the signature of localized buckling.

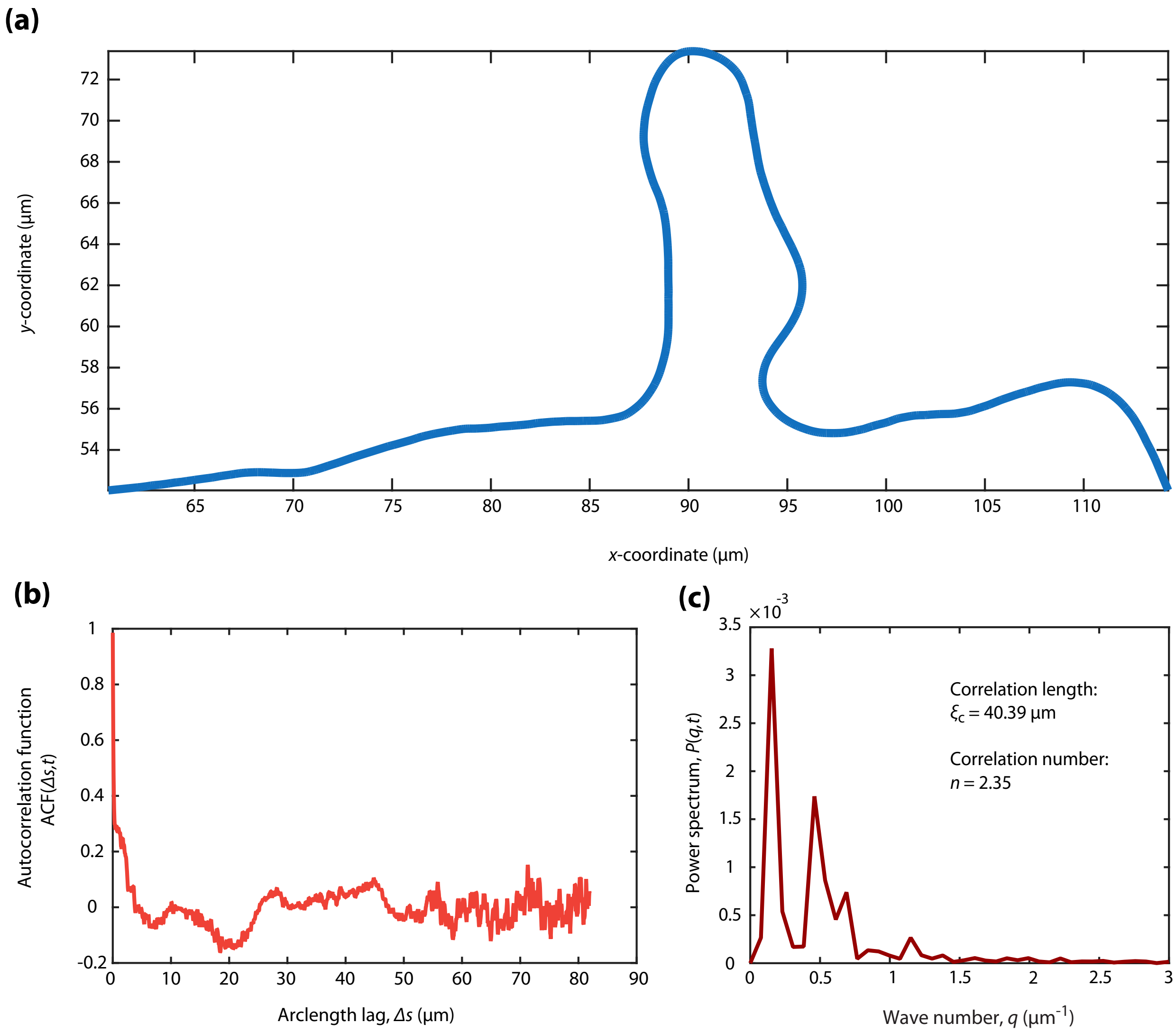

Fig. S10. Nonmotile *P. aeruginosa* chains exhibit localized buckling. (a) Centerline configuration of a buckled chain in $18\,\mathrm{w/w\%}$ DSCG. **(b)** Curvature autocorrelation function $\mathrm{ACF}(\Delta s, t)$ of the chain in **(a)**. **(c)** Power spectrum $P(q, t)$ of the chain in **(a)** after the onset of buckling. The correlation length is $\xi_\mathrm{c} = 2\pi/q_\mathrm{c} = 40.39\,\mathrm{\mu m}$, giving a correlation number $n = L/\xi_\mathrm{c} = 2.35 > 1$.

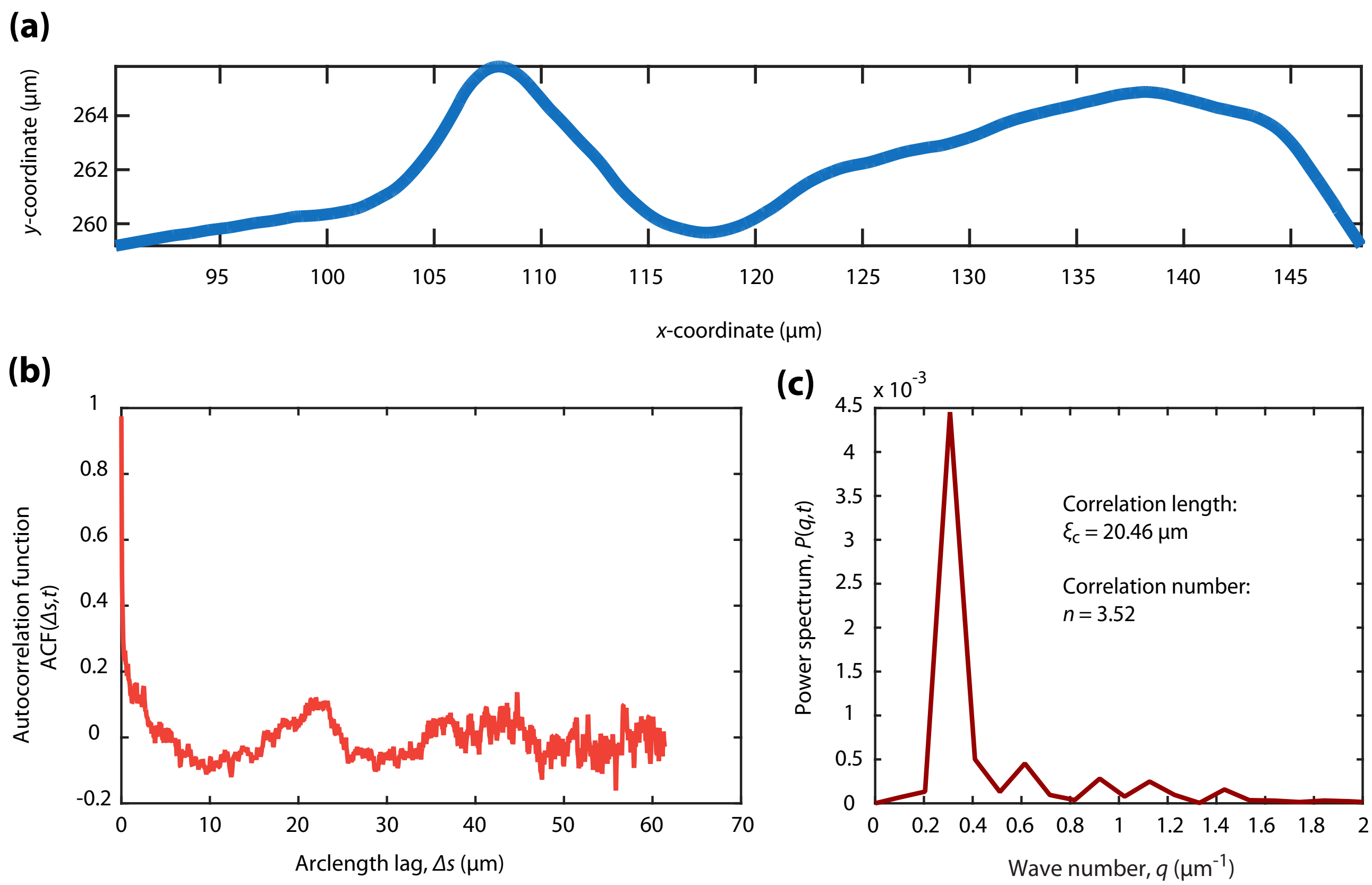

Fig. S11. Nonmotile *P. aeruginosa* chains exhibit localized buckling. (a) Centerline configuration of a buckled chain in $18\,\mathrm{w/w\%}$ DSCG. **(b)** Curvature autocorrelation function $\mathrm{ACF}(\Delta s, t)$ of the chain in **(a)**. **(c)** Power spectrum $P(q, t)$ of the chain in **(a)** after the onset of buckling. The correlation length is $\xi_\mathrm{c} = 2\pi/q_\mathrm{c} = 20.46\,\mathrm{\mu m}$, giving a correlation number $n = L/\xi_\mathrm{c} = 3.52 > 1$.

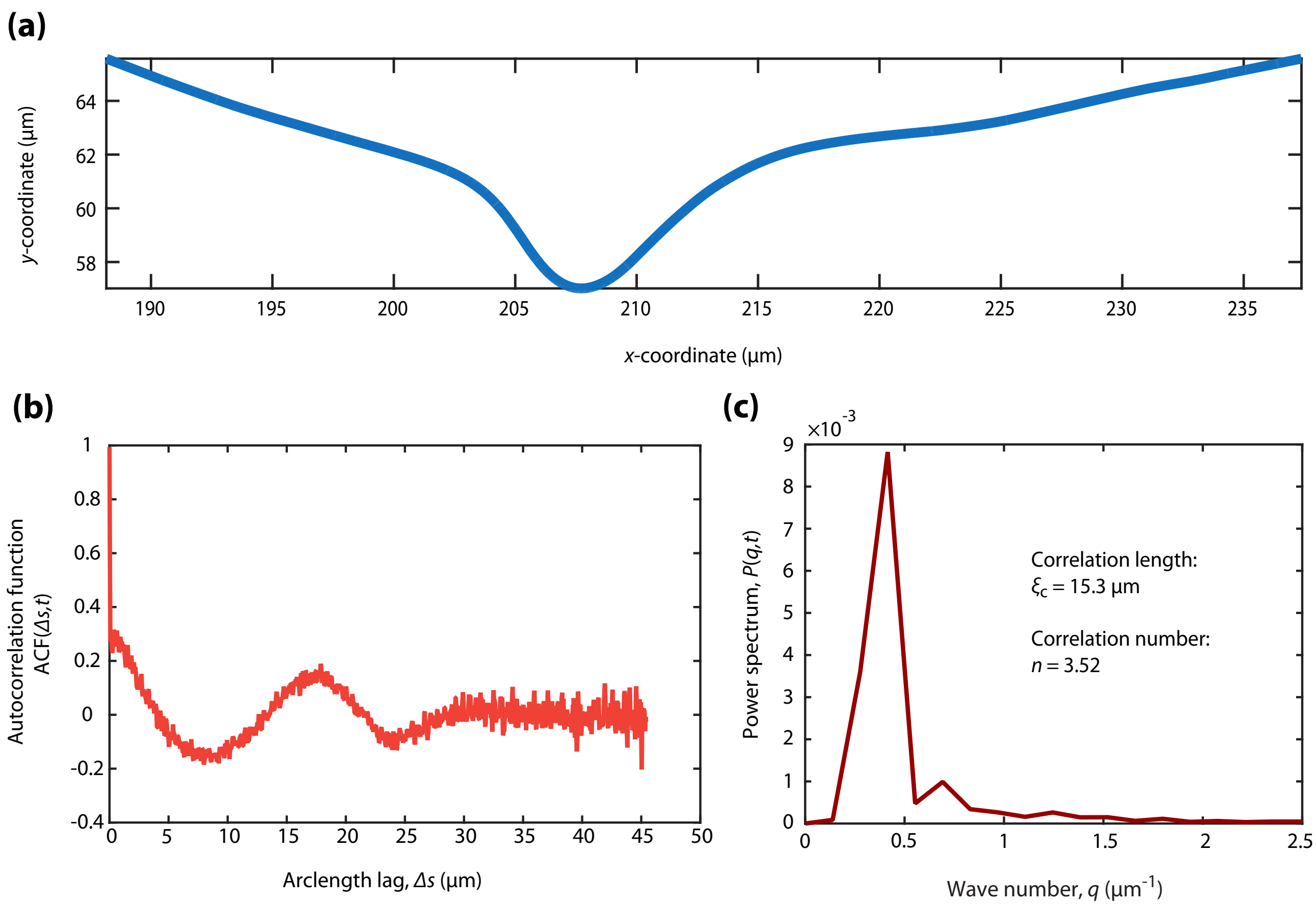

Fig. S12. Nonmotile *V. cholerae* chains exhibit localized buckling. **(a)** Centerline configuration of a buckled chain in $18\,\mathrm{w/w\%}$ DSCG. **(b)** Curvature autocorrelation function $\mathrm{ACF}(\Delta s, t)$ of the chain in **(a)**. **(c)** Power spectrum $P(q, t)$ of the chain in **(a)** after the onset of buckling. The correlation length is $\xi_\mathrm{c} = 2\pi/q_\mathrm{c} = 15.3\,\mu\mathrm{m}$, giving a correlation number $n = L/\xi_\mathrm{c} = 3.52 > 1$. Together with Figs. S9–S11, this analysis confirms that localized buckling $(n > 1)$ is robust across the bacterial species tested.
